# The Learn Together programme (part A): co-designing an approach to support patient and family involvement and engagement in patient safety incident investigations

**DOI:** 10.3389/frhs.2025.1529035

**Published:** 2025-03-26

**Authors:** Jane K. O’Hara, Lauren Ramsey, Rebecca Partridge, Chris Redford, Siobhan McHugh, Gemma Louch, Penny Phillips, Laura Sheard, Ruth Simms-Ellis, Justin Waring, Joe Langley

**Affiliations:** ^1^ The Healthcare Improvement Studies (THIS) Institute, University of Cambridge, Cambridge, United Kingdom; ^2^ Yorkshire and Humber Patient Safety Research Collaboration, Bradford Institute for Health Research, Bradford, United Kingdom; ^3^ Lab4Living, Sheffield Hallam University, Sheffield, United Kingdom; ^4^ Nifty Fox Creative, Barnsley, United Kingdom; ^5^ School of Humanities and Social Sciences, Leeds Beckett University, Leeds, United Kingdom; ^6^ School of Healthcare, University of Leeds, Leeds, United Kingdom; ^7^ Patient and Family Advisory Group Member – Learn Together programme, Leeds, United Kingdom; ^8^ York Trials Unit, University of York, United Kingdom; ^9^ School of Psychology, University of Leeds, Leeds, United Kingdom; ^10^ School of Social Sciences and Humanities, Loughborough University, Loughborough, United Kingdom

**Keywords:** patient safety, patient involvement, healthcare harm, safety investigations, healthcare litigation, qualitative research

## Abstract

**Background:**

Whilst patients and families can and do support patient safety in several ways, empirical evidence for the specific impact of involvement in patient safety incident investigations and their outcomes, has been limited, with little information about how to undertake involvement meaningfully.

**Aim:**

We aimed to (i) develop a set of common principles to guide involvement of patients and families in patient safety incident investigations; (ii) develop a working programme theory for how these might be enacted; (iii) co-design guidance to support the meaningful involvement of patients and families in patient safety incident investigations.

**Methods:**

We synthesised three existing data sets (a literature review, a documentary analysis of incident investigation policies and 42 interviews with patients, families, lawyers, incident investigators, and healthcare staff) relating to patient and family involvement in incident investigations. Ten common principles and a working programme theory were drafted. Within a convened co-design community, we then developed guidance for patients, families, staff, and investigators in local NHS Trust and national investigations, via a series of workshops.

**Findings:**

We developed ten ‘common principles” and a working programme theory for an approach that might support meaningful patient and family involvement in incidents investigations. Based on these principles and the programme theory, we co-designed guidance to be used within NHS Trust and national investigations of harm that follow patient safety incidents. The guidance includes information, resources and tools to enable better understanding and practice, from the perspective of patients, families, investigators and staff, on how to be meaningfully involved.

**Conclusions:**

Our ten common principles and co-designed guidance emphasise two key things. First, that organizational learning is not the only desired outcome for incident investigations, with patients, families and staff reporting the need for restoration and repair. Second, that investigations can be part of reparation, but when it fails to address the needs of stakeholders arising from investigations, it can compound the harm of the original incident. As a result, we juxtapose existing theories, and illuminate new insights, proposing a theory of “restorative learning”. We see design as an ongoing phenomenon—the guidance is our current iteration, and we learnt several valuable lessons about doing co-design.

## 1 Introduction

Growing evidence has demonstrated that patients and families can and do support patient safety in several ways (1, 2), including identification of safety concerns and incidents (3) that other error detection methods (e.g., staff reports, case note review) may not access (4). Some evidence suggests that patients and families can specifically contribute to organisational learning as part of incident investigation processes. For example, two US studies explored experiences of patients and families, and their ability to comment on factors contributing to the incident (5, 6) finding they were able to identify three contributory factors on average. These data were used to develop a tool (IMPACT) for structuring conversations with patients and families after an incident, gathering information for investigations (7). However, the tool appears to not have been implemented or tested in healthcare organisations. Another US study proposed a process for involving patients and families in root cause analysis (8), but this was not evidence-based nor tested in practice.

Despite the building evidence and policy directives in the UK highlighting the importance of patient and family involvement in patient safety initiatives and incident investigations [e.g., (9)], empirical evidence for the impact of involvement upon investigations and their outcomes, is limited. In addition, whilst exhortations for involvement are widespread, there is little detailed information about how to involve patients and families in investigations of incidents meaningfully.

**Figure 1:**
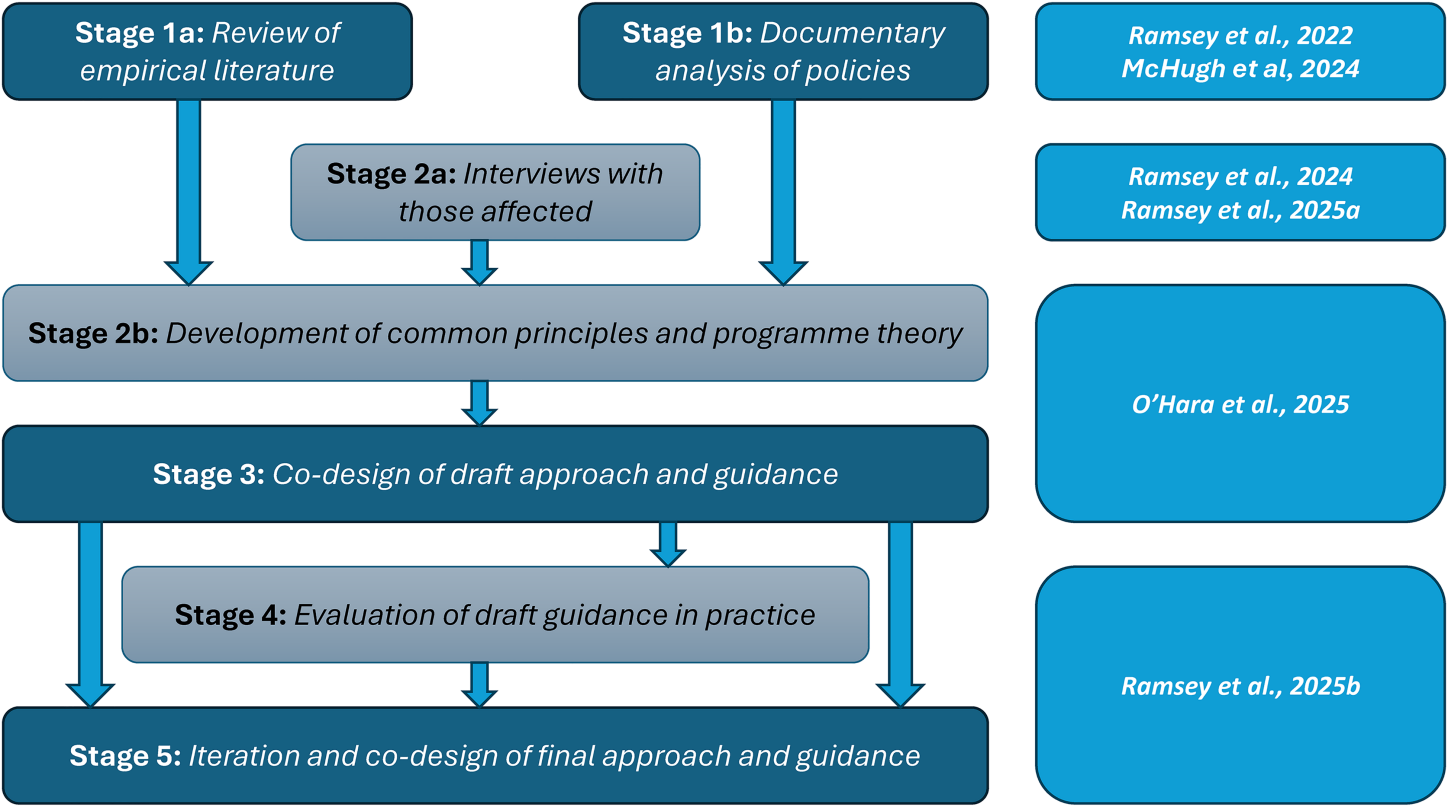
Overview of the Learn Together programme (https://learn-together.org.UK).

The Learn Together programme, funded within the UK by the National Institute for Health and Care Research (NIHR), aimed to meet this need by developing practical guidance to support organisations and investigators to undertake involvement. The Learn Together programme (https://learn-together.org.uk) was a five-year, multi-stage programme of research. Figure 1 provides an overview of the programme. Stages 1a and 1b of this programme sought to establish the current landscape of involvement in incident investigations, via a literature review (10), and a documentary analysis of current incident investigations policy within English NHS Trusts (11). The literature review did identify a number of relevant interventions tested within the US designed to support disclosure, discussion and proactive compensation where standards of care are not met, such as Communication Resolution Programmes (CRP's) (12–15), the Disclosure, Apology and Offer Model (DA&O) (16) and Recognise, Respond and Resolve (3R's) (17). Stage 2a extended this exploration of the landscape, with a large interview study of key stakeholders—patients, families, healthcare staff, legal representatives, and those investigating incidents within NHS Trusts (18, 19).

Collectively, this work emphasised how it was essential that processes after harm considered both clinical and emotional aspects of care. However, it was also clear that evidence was not translating into practice routinely in the UK, and significant gaps in the evidence remained. A further important finding from this exploration of the current landscape, was that it had historically not drawn on, or sought to explicitly develop, theory. This is perhaps understandable, when the topic is one that has traditionally been driven by policy directives and patient or family advocacy. However, it is a significant limitation of current empirical and practitioner-focused literature, particularly when used to develop or evaluate mechanisms to improve involvement and engagement following harm.

One well-known safety theory that might provide a useful lens on this topic—what might be seen as a “grand theory” (36)—is the organizational accident model (20). This framing posits that patients and families can provide valuable information about patient safety incidents, that, if acted on, could reduce the likelihood of future incidents. However, this theoretical lens is arguably limited in its focus to the transactional elements of processes following healthcare harm—principally organizational learning—and much less on the relational elements of investigations. A second lens on this topic, which has been growing in interest over the past five years, is the possibility of alternative restorative approaches to responding to harm events (21). In contrast to the systems safety approach described above, which emphasizes organizational learning and prevention of future harm as the motivation for investigations, restorative approaches focus on those who have been harmed or who are affected by the harm, what their needs are, who is responsible for meeting those needs, and only after these are addressed is the question of prevention of future harm raised (22). Interventions which focus on reconciliation after harm were identified in the literature review (10) and included IMPACT (7), CRP's (12–15), DA&O (16) 3R's (17) and next-of-kin involvement (23, 24), which had been largely considered in the context of the US healthcare system, as well as Norway. Given the current evidence base, understanding of this phenomenon may benefit from being considered together with additional theoretical lenses such as restorative approaches in a UK context (25).

A further conclusion from our exploration of the current landscape was that both empirical and practitioner-focused approaches to involvement had often been developed from one particular perspective—either patients or staff. This was despite Langer et al. (26) highlighting how patients and healthcare staff can effectively collaboratively learn in the context of patient safety incidents and prevention. What was evident was a lack of systematic attempts to bring together the diverse groups of people with a stake in investigations and their conduct, to design something that would work within the complexity of the systems into which they would be adopted. For this reason, the Learn Together programme centred co-design, from the development of the application for funding (collaborating with patients, families, and healthcare staff and managers) through to a longitudinal, deliberative co-design process at its core.

Co-design is increasingly being used in health research involving patients, caregivers, healthcare professionals and policy makers. In design research literature, it is described as “collective creativity”, with designers and people not trained in design working together in the design development process (27). The involvement of stakeholders, particularly end users, is based on two fundamental justifications—one democratic (“Nothing about me without me.”) and the other pragmatic (“I hold valuable contextual knowledge about how things work or don't work in my context/practice”) (28, 29). Including all stakeholders in developing interventions, services or policies is likely to account for the complexities of the system, pick up on nuances not covered by more generalisable evidence, and give it a greater chance of adoption and impact.

Bringing these strands together, this paper presents the detailed methods and findings of *Stage 2b and Stage 3* (see Figure 1) of the Learn Together programme. We present the integration of data emerging from our exploration of the current landscape—and the generation of a set of common principles and working programme theory for the proposed approach—and the engagement with key stakeholders in a design process based on these principles and programme theory, to develop draft guidance to support the more meaningful involvement of patients and families in patient safety incident investigations.

Our research objectives were to:

(1) Develop a set of *common principles* to guide involvement of patients and families in patient safety incident investigations;
(2) Develop a *working programme theory* for how these might be enacted in healthcare organisations;
(3) Based on these principles and working programme theory, *co-design guidance* to support the meaningful involvement of patients and families in patient safety incident investigations.

## 2 Methods

### 2.1 Ethical approval

Ethical approval was not necessary for this work. However, relevant ethical approvals were gained for the primary data gathering that informed the work [detailed in (18, 19)].

### 2.2 Study design

A mixed methods design was used, combining abductive secondary analysis of existing data [detailed in (10, 11, 18, 19)], and a co-design process.

### 2.3 Developing common principles and a working programme theory

This phase pertains to Stage 2b of the Learn Together programme (Figure 1). Between January and April 2021, we conducted a three-phase analysis and synthesis of three existing data sets. This included (i) a review of the evidence surrounding patient and family involvement in serious incident investigations (10), (ii) a documentary analysis of NHS Trust policy surrounding patient and family involvement in serious incident investigations (11) and (iii) an interview study relating to experiences of involvement in incident investigations with patients, families, investigators and staff (18, 19). We started with inductive, and then moved to abductive analytical approaches, to create meta-level findings. Analysis was both descriptive and conceptual. The descriptive, inductive analysis allowed for an understanding of “what participants said” whilst a higher, conceptual level analysis provided an interpretation of what this meant for the involvement of patients and families in serious incident investigations.

#### 2.3.1 Phase 1: inductive analysis

To prepare for the synthesis of the findings, short descriptive reports were created for each of the data sets—the evidence review (10), the documentary analysis of policies (11) and the interview study (18, 19). For the interview study data, due to the volume of interviews and purposive sampling by group, a short report was created for three groups represented (patients/families, staff, investigators). Reports were created inductively by the lead researcher on each study, and as such had variable structures, before being shared within the team for refinement and agreement ahead of synthesis workshop 1.

#### 2.3.2 Phase 2: abductive analysis

Analysis then moved to an abductive phase to create common principles. Abductive analysis is a qualitative approach that involves an interplay or middle ground between inductive and deductive type reasoning. It resembles an iterative cycle of analytical reasoning, in that ideas and themes identified within empirical data are used to develop concepts and propositional statements, which are then related back to the existing literature and theory to determine whether they are plausible and whether they confirm, extend or question the existing evidence. This then generates additional questions and theoretical ideas about the field of enquiry that are then examined through further analysis of the data (30).

#### 2.3.3 Analysis procedure

**Figure 2:**
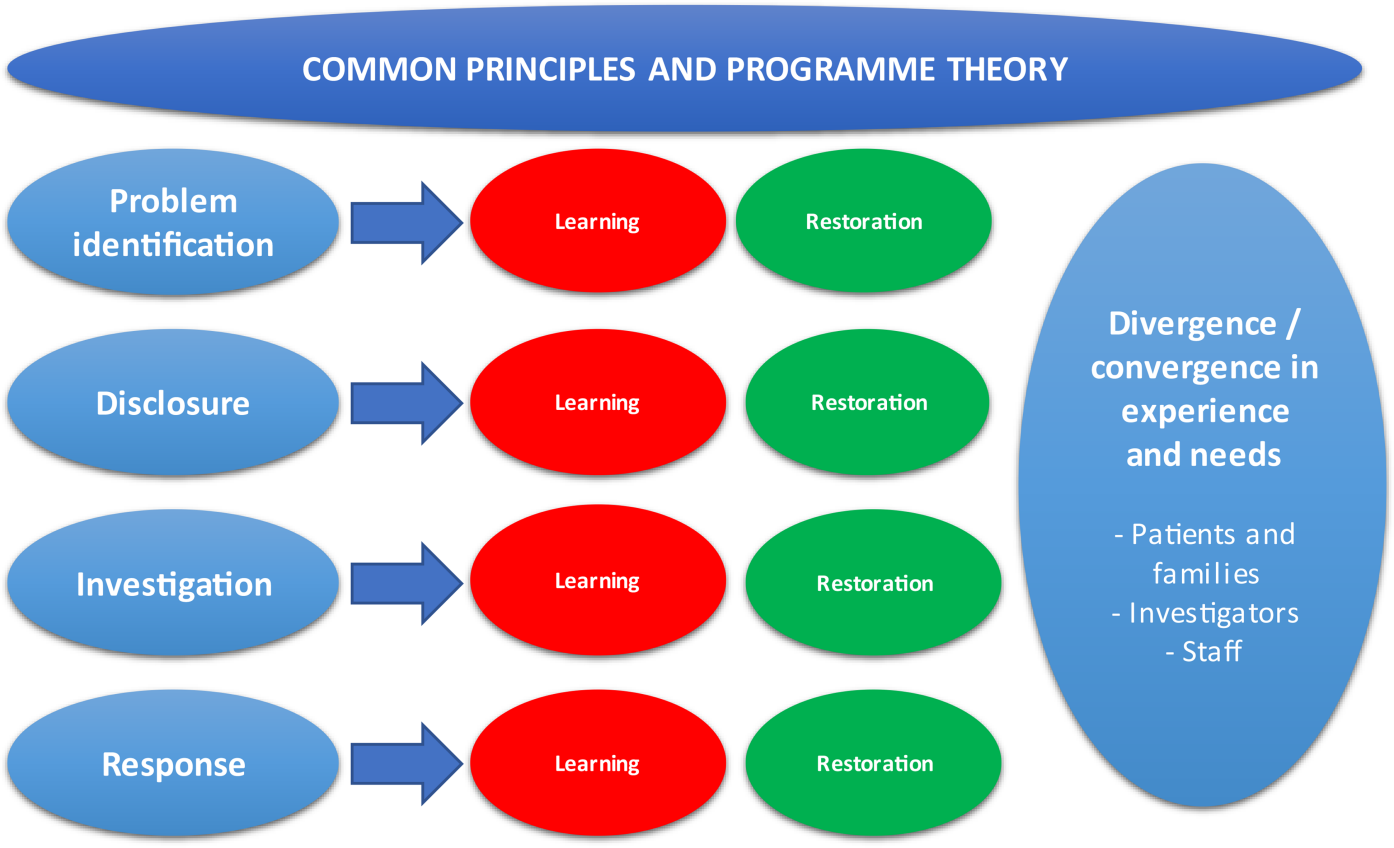
Abductive analytical framework.

We created an analytical framework based on the two theoretical lenses—broadly conceived as 'systems safety' and “restorative” approaches (illustrated in Figure 2). First, we were interested in the role for, and experience of, patient and family involvement in the creation of organizational learning. Second, we were interested in the needs that are created for patients and families due to the experience of a patient safety incident, but importantly, also the experience of the investigatory process itself, and the potential for that experience to compound the initial harm and dictate further action (such as complaints and litigation). In addition to these two theoretical lenses, we were also interested in a third, practical aspect of the investigatory process—namely, how the investigation is experienced as a temporal phenomenon. In particular, we were interested in whether the needs for learning and repair were different across the phases of an investigation, and across the different 'stakeholder' groups who are involved in the process and practice of investigations: patients and families, healthcare staff and investigators.

The research team met in several intense analysis sessions to conduct the abductive analysis. Based on the content of the short reports developed from the inductive analysis and researchers’ own knowledge of the field, the analysis team discussed each theoretical lens in relation to the empirical data. This involved reflecting on the convergence and divergence between the three analytical lenses—systems safety, restorative approaches and the temporal nature of an investigation From this intense analytical work, the abductive analysis framework (see Figure 2) was constructed and findings were written up which paid attention to the core constructs of the common principles.

#### 2.3.4 Phase 3—synthesis

This phase ran concurrently with the first two, supporting an ongoing synthesis of the inductive and abductive analysis, to more fully develop draft common principles for meaningful involvement, and a programme theory within which they could be framed. Three workshops were attended by the research team, undertaken between March and April 2021. These workshops were bookended and punctuated by the analysis phases, which provided data for each workshop in turn. All workshops had an agenda and desired outputs agreed in advance.

### 2.4 Co-designing new guidance

**Figure 3:**
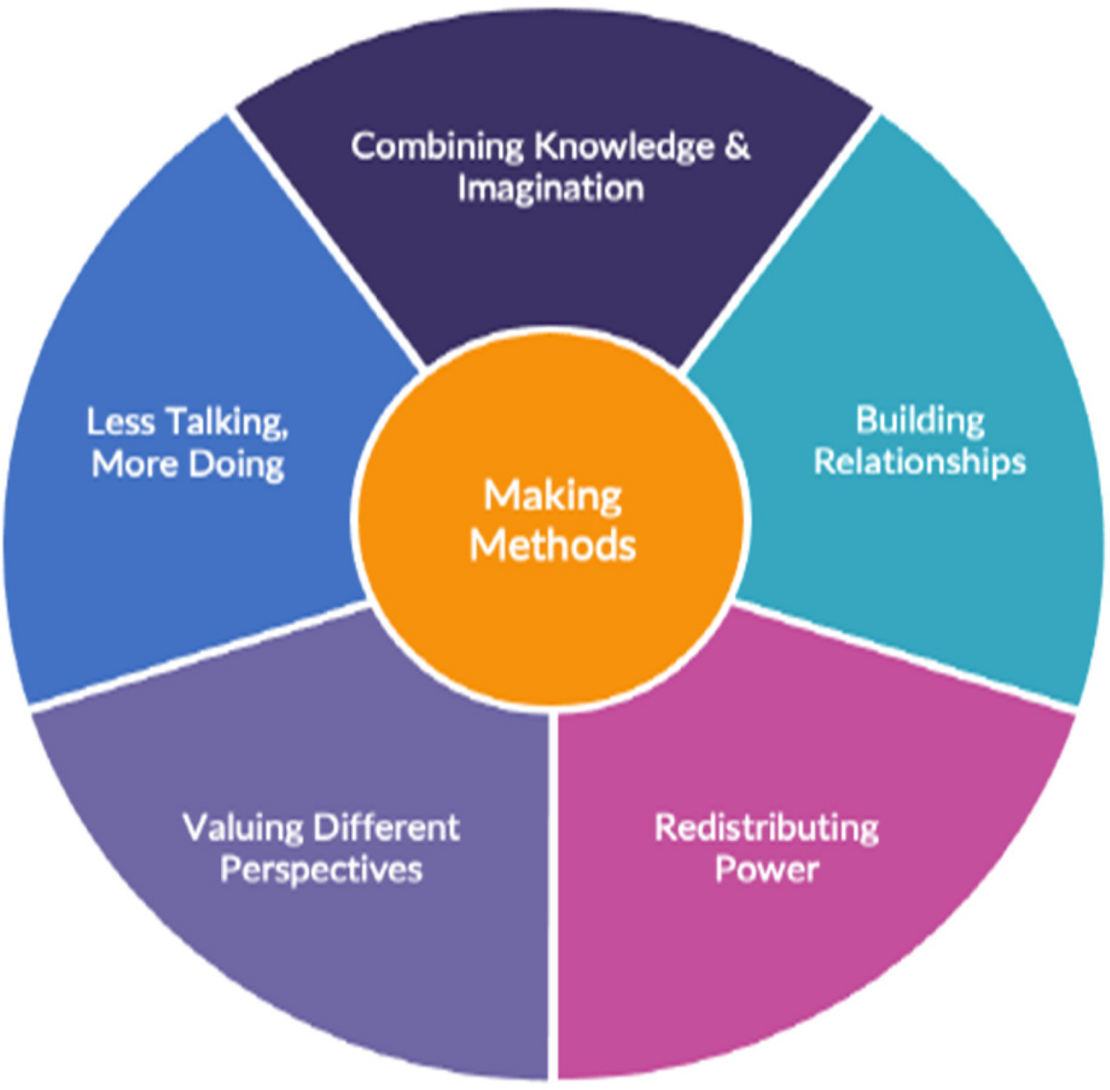
Our co-design framework.

This pertains to Stage 3 of the Learn Together programme (Figure 1). The co-design process was informed by the UK Design Council Double Diamond for Innovation (31). We also drew on other frameworks (32, 33), and combined these with a framework that has been developed by co-authors and their wider team (Lab4Living: Figure 3) to guide their co-design practice.

#### 2.4.1 Developing the co-design community

We formed a virtual “Co-design Community” with representation from patients, family members, healthcare staff, investigators, family liaison officers, policy representatives, legal staff, academics, researchers, and designers. Outside of the core research and design team, this community was drawn together by two principal mechanisms: (i) known stakeholders (such as national policy makers and representatives from patient advocacy organisations) were invited directly via personalised emails; and, (ii) snowball sampling of other stakeholder groups (such as healthcare staff, and legal representatives) was undertaken via recommendations from included community members. All patient and family representatives in this community were either members of the programme's Patient and Family Advisory Group (see https://learn-together.org.uk for more details), people recommended to us through this group, or participants from the Stage 2a interview study.

As detailed below, part of the co-design process broke into smaller workstreams to focus on different contexts. Some co-design partners attended multiple workstreams. The mental healthcare workstream included 21 co-design partners, the acute care workstream had 30 co-design partners, and the national independent investigatory body workstream had 22 co-design partners. The subsequent four development sessions were drop-in open invitation. Approximately 17 people attended each of these.

The design team (JL, RP, CR) and research team (JOH, LR, SMcH, RSE) collaborated throughout this process ensuring research evidence was fed into the co-design activities, and feasible, actionable outcomes that could be evaluated were derived.

#### 2.4.2 Planning the co-design interactions and activities

Co-design activities were structured around two large stakeholder events “bookending” a series of three co-design workshops that ran in three parallel workstreams. The three parallel workstreams reflected different contexts of incidents and investigations: (i) in acute care settings, (ii) in mental healthcare settings, and (iii) in the national independent investigatory body (Healthcare Safety Investigation Branch: HSIB). Draft outlines for co-design sessions were prepared by the design team following discussion with the research team. These outlines were iteratively refined with the research team and the patient and family advisory group.

Co-design should include all perspectives, and the co-design activities and outcomes should be based on collective experiences *and* on evidence obtained from research. To ensure fidelity to this approach, we wanted to (a) build and sustain relationships and trust between all co-design partners, the research team and the design team; and (b) provide the evidence from our research phase in an accessible format that enabled people to make sense of it in relation to their own experiences and give them an insight into others' experiences. In the planning and preparation for the co-design interactions and activities, we were mindful of the emotional investment for all, the probability of oppositional viewpoints and the potential for wide differences in expectations about what could or should change.

#### 2.4.3 Curating the flow of research evidence into the co-design process

A common criticism of co-design is that the product or outcome of a co-design process is likely to be heavily biased by the specific individual experiences of the co-design partners. However, it is important that co-design is informed by both research evidence and the specific experiential evidence of co-design partners. A challenge here is the possibility of these different evidence 'sources’ residing with different people, or being seen to have different “power”, both of which can result in oppositional or competitive scenarios. Therefore, we deliberatively set out to share the research evidence with all co-design partners ahead of the process, so that everyone could understand and value both research and experiential evidence. To do this, the common principles, working programme theory and evidence from the three data sets (Stages 1a, 1b and 2a) were synthesized into rich descriptive narratives which were converted into a sense-making activity called the “Rebuilding Investigations Kit” (34) that could be posted to co-design partners to complete before the co-design process. The activity is explained more fully further on, but in summary the rich-descriptive narratives were fragmented (onto a set of cards) and give alternative narrative pathways. The sense-making dimensions was based on principles of narrative assembling, and decision-making narrative pathway options.

#### 2.4.4 Co-design interactions and activities

**Figure 4:**
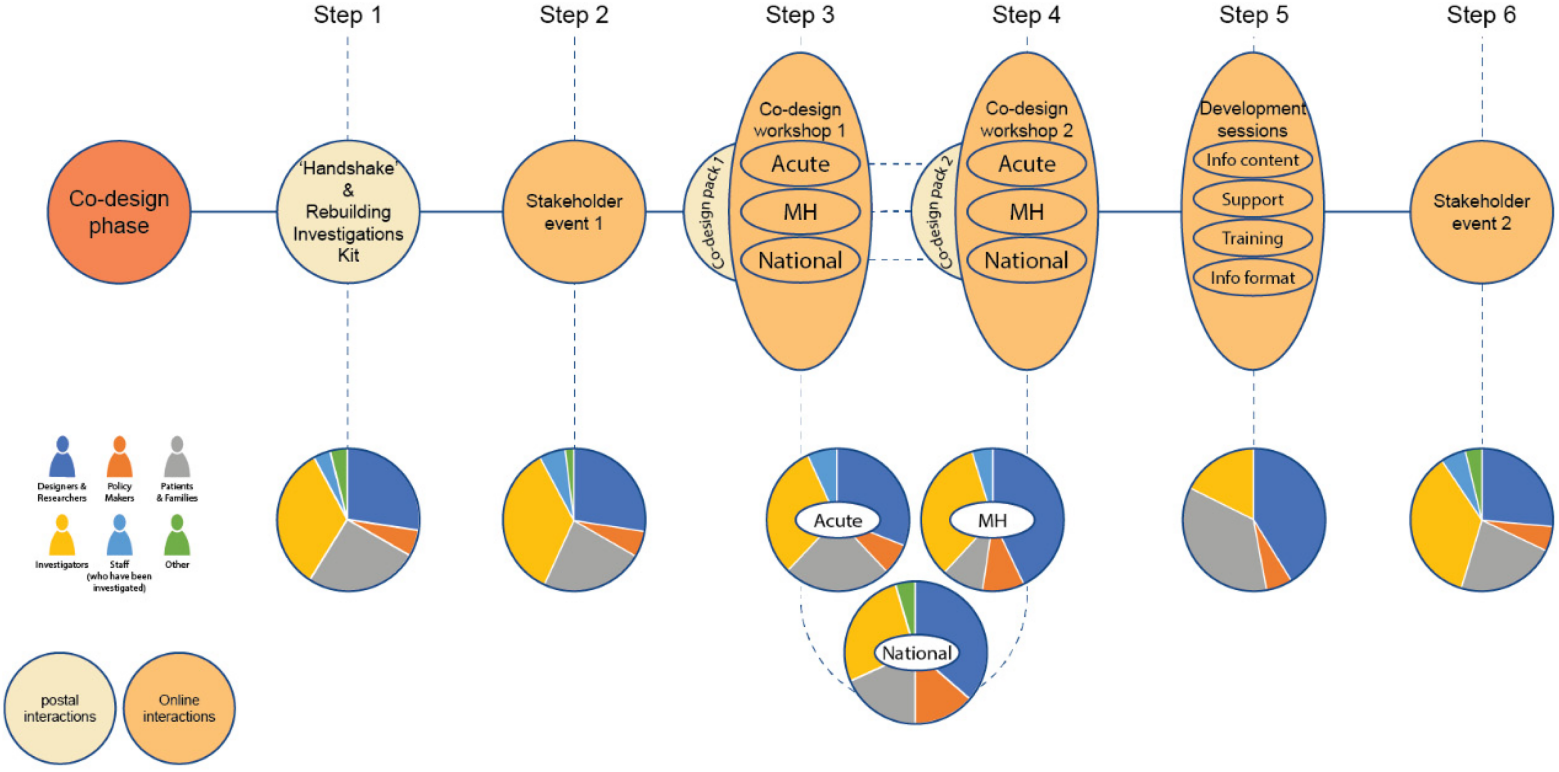
Co-design process and activities.

Figure 4 illustrates the initial co-design process, activities and proportions of different stakeholders in each. We describe each step in more detail below.

Step 1: “Handshake” & Rebuilding Investigations Kit.

Step 1 included two posted interactions: The “Handshake” and “The Rebuilding Investigations Kit”. Both kits were designed to fit into a letterbox sized, A5 or A4 boxes, printed in colour and professionally bound. The obvious care and unique content implied a significant time investment, demonstrating we valued the process and our partners.

The first posted interaction—called “*The Handshake”*—comprised the “welcome booklet” (Supplementary Material S1), creative origami activity, and tea bag. In calling this “*The Handshake”* we tried to recreate aspects of meeting in-person for the first time—greeting people, identifying the team, and welcoming them into a community. We attempted to re-create elements of this via design decisions about content and form of this pack. The tone of the booklet was simple, accessible, and friendly. It included team photos to humanise the team, and presented the process to help set expectations. The origami activity was deliberate; we wanted people to use their hands to make something. Such activities help to move ideas beyond words into tangible representations, helping people think about them differently. This origami helped familiarise people with methods that would be used in the co-design process. The tea bag, along with the postal and boxed nature of this interaction were intended to mimic gift experiences. In relation to the Lab4Living co-design framework, this pack started the process of building relationships, sharing power, and getting people “doing things”.

The “*Rebuilding Investigations Kit”* [see (34)] was sent with the intention that co-design partners would do this as a reflective, individual activity, taking approximately one hour, as a way of understanding the research evidence. The activity described a “past-present-future” narrative where the *past* was a patient safety incident, the *present* was the investigation of that incident, and the *future* described the longer-term impacts of the incident and the investigation on the various characters in the narrative. The narrative was fragmented into a deck of cards, so it had to be physically and cognitively re-assembled into a coherent whole. Collectively, the cards reflected fragments of an overarching narrative designed to facilitate a deeper understanding of the common principles, programme theory and research evidence. At key points through the past, present and future narrative steps, the co-design partners were prompted to record their reflections.

The activity required these cards to be laid out on a large paper activity mat, printed with separate “channels” corresponding to each character in the incident narrative (patient, the patient's adult son, the nurse, the ward manager, the institution). Image examples are available online (34). Once laid out this way, differences in how the event was perceived and experienced could be seen in relation to each other. The “present” (investigation) phase of the activity, required the person interacting with the kit to adopt the role of investigator and make choices that modified the narrative of the investigation, changing evidence they gained access to and ultimately their understanding of the incident. This highlighted how some perspectives can become 'silenced', distorting the narrative, and resulting in a form of narrative (epistemic) injustice. The final phase of the kit required the person to lay out the longer-term impacts of the incident and investigation for each character in the narrative.

Feedback indicated that some co-design community members completed the activity with family, and some repeated it, making different investigator choices and reaching different conclusions. For example, one member concluded that the likely “cause” of the incident was a prescription error, while others concluded the significant factor was the pharmacist who had verbally identified the prescription error, but had not challenged it. In relation to our co-design framework, this activity communicated research knowledge and built appreciation for different perspectives our co-design partners would bring. The kit continued the theme of “less talking and more doing” by encouraging co-design partners to externalise thoughts, engaging with the kit's physical components.

Feedback from co-design partners stated this activity broadened their understanding in “non-threatening ways”, perhaps best summed by these quotes from co-design partners, who were family members of people who died while in NHS care:

"It was the first time [in 15 years] I had felt sympathy for staff.”

“It was a very disarming way of enabling me to ’see’ different perspectives.”

Step 2: Stakeholder event 1.

The community met for the first online stakeholder event in April 2021, to share and iterate the ten “common principles”, discuss the research findings presented within the Rebuilding Investigation Kit, and define a common goal. This event (along with preceding activities in step 1) brought people together, built relationships and created conditions to take their own place within the co-design team.

An independently commissioned company facilitated both stakeholder events (steps 2 and 6), enabling the research and design team members to sit “alongside” co-design partners, all of us responding to activities and prompts in the same way, and reduce any perceived power imbalances between the co-designers and researchers, and co-design community members. In relation to our co-design framework, this continued the process of building relationships, introducing and examining the extant knowledge (research evidence), and sharing of power (by sharing knowledge).

Steps 3, 4 and 5: Co-design workshops 1 and 2, and development sessions.

Six workshops focusing on needs and ideas generation, were undertaken between May and August 2021. These comprised two mental healthcare workshops, two acute care workshops and two national independent investigatory body workshops. Each workshop was proceeded by a co-design kit posted to participants before workshop 1 and 2. Each kit contained individual reflective exercises supporting co-design partners to gather their thoughts in preparation for the next online workshop.

The workshops were followed by four development sessions iteratively refining shortlisted ideas. We brought the three workstreams together for these to compare ideas. We looked at similarities and differences to understand whether they had a common base or were fundamentally different due to context. The co-design group recognised that there were similarities, and important variations between mental healthcare and acute contexts. For example, investigations in mental health contexts often related to suicide in the community. It also seemed that mental health investigations frequently involved a greater number and diversity of stakeholders such as social care providers, primary care providers, police, education providers, employers, coroners etc. However, it was decided to deliver the same solution in both settings, and evaluate these distinctions as we did not, at that stage, understand these differences sufficiently for a design response. The maternity investigations led by the national independent investigatory body, had very different resources, a more consistent approach to investigations, dedicated investigators, and family engagement staff, and had previously conducted work developing approaches to engage patients and families in investigations. These important distinctions lead to solution variations in the national workstream.

##### 2.4.4.1 Initial identification of solution concepts

These broad areas of solution concepts arose from the workshops:

1. Support café
2. Information:
  a. Physical/printed
  b. Digital/website
  c. Online chat/advice
3. Investigation training resources (for investigators and organisations about involving patients and families in investigation processes)
4. Improvement centre (collating and implementing recommendations)
5. Team approach to investigations (support for investigators)
6. Patient-led investigations

These labels encompass complex concepts. For example, the topic idea of 'Support Café' was not a physical café, but rather conceived to be a “one-stop-shop” menu of support (practical, physical, emotional, cognitive, legal, “technical”) for patients and families experiencing an incident and subsequent investigation, and those who had historically experienced such events. “Café” represented informality and even conviviality as opposed to formal support provision (e.g., professional counselling, guidance etc). This did not exclude professional support but emphasised peer-to-peer and lived-experience support.

Ideas around “Information” were separated into different media or channels to accommodate differences in design, production, implementation, and on-going maintenance and associated implications, costs, and resources.

##### 2.4.4.2 Concepts taken forward and developed

From the co-design outset, we stressed this project was a starting point. Not everything could or should be addressed within the current project scope. In Stakeholder Event 2, we asked participants to use the following criteria to assess and select ideas to take forward: (i) **perceived benefit** of achieving the research aims; (ii) **chronology—**high priority ideas might require other changes first; (iii) **scope**—ideas not in the scope of the project brief were deferred for later projects; ix) **feasibility** within the current system.

Of the broad areas above, information (all three categories) and investigation training resources were prioritized to be developed further. There was a broad consensus from all co-design partners that unless all stakeholders (investigators, staff being investigated, patients/families and organisations) were better informed, then all other areas of development would be compromised. This rationale prioritised both information and training. The co-design process, which was fully documented using an accessible online collaboration tool (Miro), equipped co-design partners to understand these parameters and make an informed choice on the filtering of ideas at this stage.

Step 6: Stakeholder event 2.

In September 2021, Stakeholder Event 2, the outputs (still in draft design form) were presented to the co-design community for critical review and comments.

##### 2.4.4.3 First draft of guidance development

The core research team compiled content for the new guidance, bringing together the co-designed ideas with the evidence, policy requirements and technical specifications such as the correct terminology. This content was designed by the design team (RP, CR) into prototypes and shared for discussion with the research team, and other key stakeholders, in particular members of Patient and Family Advisory Group and Staff Advisory Group. This became an iterative cycle of content and design development, gathering feedback, and making changes. Due to the existing support materials and approaches already in place for HSIB maternity investigations, we developed content specific for this setting. One researcher (SMcH) undertook a mapping session with HSIB collaborators, to understand their current approach relative to our proposed approach. The gap identified was that families were not “active participants” in HSIB's current approach. To meet this gap, we developed a Family Reflective Booklet, and investigator training to support using this within their current approach.

All draft guidance were ratified by members of the patient and family, and staff advisory groups, as well as the research steering group. The guidance supported a new process for the involvement of patients and families through patient safety incident investigations (see Box 1 for cover illustrations). For acute and mental healthcare settings, this comprised (i) an investigator guidance booklet, (ii) investigator training material and content, (iii) an investigation record (for investigators to use for each investigation) and, (iv) a patient safety incident investigation information guide for patients or family members. For the national independent maternity investigations, this comprised a new Investigation Reflection booklet and investigator briefing (learn-together.org.uk).

Box 1 **Ten common principles to support restorative learning**

1) ***Make apologies meaningful****:* rather than offer excuses, demonstrate understanding and a commitment to learn what has happened and why
2) ***Individualise your approach***: involvement should be flexible and adapt to changing needs, Set realistic expectations
3) ***Be sensitive to timing***: investigation can feel like they are happening slowly, quickly or at insensitive times. Investigators need to manage time carefully
4) ***Investigations can compound harm***: harm can happen through the experience of the investigation and how people are treated within it.
5) ***Strive for equity***: investigations allow an organization to learn, but if their agenda is prioritized over patients/staff, the process can feel discriminatory.
6) ***Provide guidance and clarity***: patients, families and even healthcare staff can all be confused by what an investigation actually entails.
7) ***Listen***: if there is a true commitment to learning, then everyone involved should have the opportunity to share their experiences.
8) ***Be collaborative and open***: it’s often much more complex than people seeking compensation or wanting to assign blame
9) ***Respect humanity***: investigations should embrace and accommodate different human responses
10) ***Accept subjectivity***: each individual will experience the same incident in different ways. No one truth should be prioritized over others.

## 3 Findings

The abductive analysis aimed to bring together and juxtapose two main theoretical lenses—a systems safety approach (20), and a restorative practice approach (21). In undertaking this analysis, we arrived at a number of key conclusions. First, that our data was not wholly accounted for by either one of these conceptual positions, and therefore an abductive, theory development approach was justified. Second, that there were divergences in the needs that different stakeholders might have, that might not always be reconciled. Third, that compounding of the initial harm (from the event) was possible throughout all the phases of activity that follow the identification of, and response to a patient safety incident. Fourth, that the experience of compounded harm was significant enough that an approach to reducing it, through more principled, meaningful and family-centred investigatory processes, was an important aim of organisational responses to patient safety incidents. Finally, we concluded that co-design was the only morally and logically justifiable method to support the development of solutions for a process which is complex, relational, and fraught with conflicting needs.

Based on the synthesis of our evidence, we developed common principles and a programme theory that reflect our abductively derived approach, which we termed “restorative learning”. This approach is sited in the assumptions that both learning and healing should be aims of any response to healthcare harm or patient safety incidents. Indeed, whilst it may not be possible to achieve no harm within the complexity of healthcare services, how organisations respond to harm when it does arise, is within their gift is.

### 3.1 Development of common principles to support restorative learning

Based on our synthesis, we developed a draft set of principles (Box 1) that could be used to develop new processes and guidance to support investigators to involve patients and families in ways that might mitigate compounded harm.

### 3.2 Development of a working programme theory

A set of evidence-based principles such as the list above are incredibly powerful at conveying the essence of a synthesised body of evidence in a manner that easily resonates with personal values and helps direct appropriate action. In short, they help to create “buy-in”. However, from an implementation perspective, the issue with a set of evidence-based principles is that they remain too conceptual, and it is challenging for people to operationalise them. Therefore, ahead of the co-design phase, we needed to integrate these principles into a working programme theory that describes how a “restorative learning” approach might be enacted to reduce the likelihood of compounding the harm for patients and their families. Ahead of the co-design, we were not seeking to prescribe the form of the new approach to the involvement of patients and families in serious incident investigations.

The working programme theory is presented as a draft narrative account (see Box 2), based on the following format: (i) the *desired outcome* of the new approach; (ii) *who* needs to be involved; (iii) *what* needs to be done; (iv) *when* should it be done; and (v) *how* should it be done.

Box 2 **Working programme theory**

**i) The desired outcome**

It was clear from the synthesis of the evidence that organisational learning was not the only desired outcome of incident investigations, for all stakeholders. Our synthesis demonstrated that patients and their families who experience patient safety incidents have a range of needs that arise, with only some of those being related directly to the incident itself—for example, physical health needs or financial compensation to support costs associated with ongoing physical or psychological health. For patients and their families, and in some ways healthcare staff too, there was a second purpose of an investigation, relating to their need to be restored or “repaired”, in part through the process of being involved in a collective sensemaking of the incident. Additionally, our synthesised findings suggest that being listened to, heard, and having their contributions and experience both valued and dignified by the investigation process, was an important part of the potential restorative impact. Importantly, our findings suggest that when patients and families are not involved in investigations in ways that they need and want to be, and where their needs are ignored or minimised, organisations can compound the initial harm. That is, the initial harm from the incident can be made worse, or in some cases eclipsed, by the harm generated from investigations that do not meet these dual needs—for learning, and for restoration and repair. Therefore, the explicit desired outcome of the approach and guidance to be co-produced, is to support restorative learning; that is, balancing the need for organisational learning with the need to minimise compounded harm.

**ii) Who needs to be involved?**

Our evidence synthesis suggests that whilst the desired outcome is to reduce compounded harm for the patient and their family, through the process of the investigation, the key actor in the new approach will be the investigator. The investigator sits at the heart of the investigation and is the fulcrum for co-ordination of involvement and engagement activity. The investigator is the principal link between the organisation and the patient and their family, following disclosure of the incident (i.e., via the Duty of Candour—a general duty to be open and transparent with people receiving care in England)

**iii) What needs to be done?**

The investigator needs to balance the needs of the organisation to learn, alongside the needs of the patient and family to get answers to their questions and feel that their experience has been given the time to be shared, listened to, and dignified. Their witness to what happened should be valued as credible information to sit alongside other sources of evidence. To undertake this important role, investigators need to be trained, have sufficient capacity, and be supported by the wider organisational system.

**iv) When should it be be done?**

This approach needs to be flexible, with the ability to person-centred; that is, able to accommodate the different needs of individual patients and their families. The nature and success of the early contacts by the organisation, and then the investigator, is likely to be perceived by the patient and their family as setting the tone for further contact through the investigation, and so should be prioritised.

**v) How should it be done?**

Without prescribing the components of the new approach ahead of the Stage 3 co-design activity, the common principles provide a starting point from which the materials and guidance would be developed. However, it is likely that information for the patient and family to support their navigation through the novel (to them), potentially retraumatising, and often confusing investigation process, will be an important component. Further, the approach needs to recognise the relational nature of investigations, and that they are not just “fact-finding” processes to establish and objective “truth”. The tensions involved in reconciling different data sources and perspectives on the same event need to be recognised and made visible for all involved. Finally, the new approach needs to prioritise and promote transparency and seek to create processes that lead to equity in outcomes.

### 3.3 Co-designing a new approach to involvement in investigations

Based on the common principles and working programme theory, we co-designed a new process and set of guidance and resources to support those undertaking investigations to involve patients and their families in local and national investigations of harm, in ways that might support restorative learning. Critically, our intention with working with all stakeholder perspectives was to ensure this new process and guidance could be used flexibly, in ways that accommodate contextual variation, rather than dictate a prescriptive approach.

The draft guidance centred around two key documents: a guidance document for investigators, and a guidance document for patients and families. These documents were designed to work together, following a similar structure. Both guidance documents were based on a temporal ordering of the investigations process, but using terms that still support some flexibility (see Box 3, and Supplementary Material S2 and S3). The investigator guidance provided a step-by-step guide to involving and engaging patients and families in investigations, using prompts and checklists. The guidance outlines how to provide both procedural and emotional support for those affected, to help to equip them with knowledge to become involved and to access the support they might need.

Box 3 **Outline draft guidance (also see Supplementary Material S2 and S3)**

Investigator Guidance 
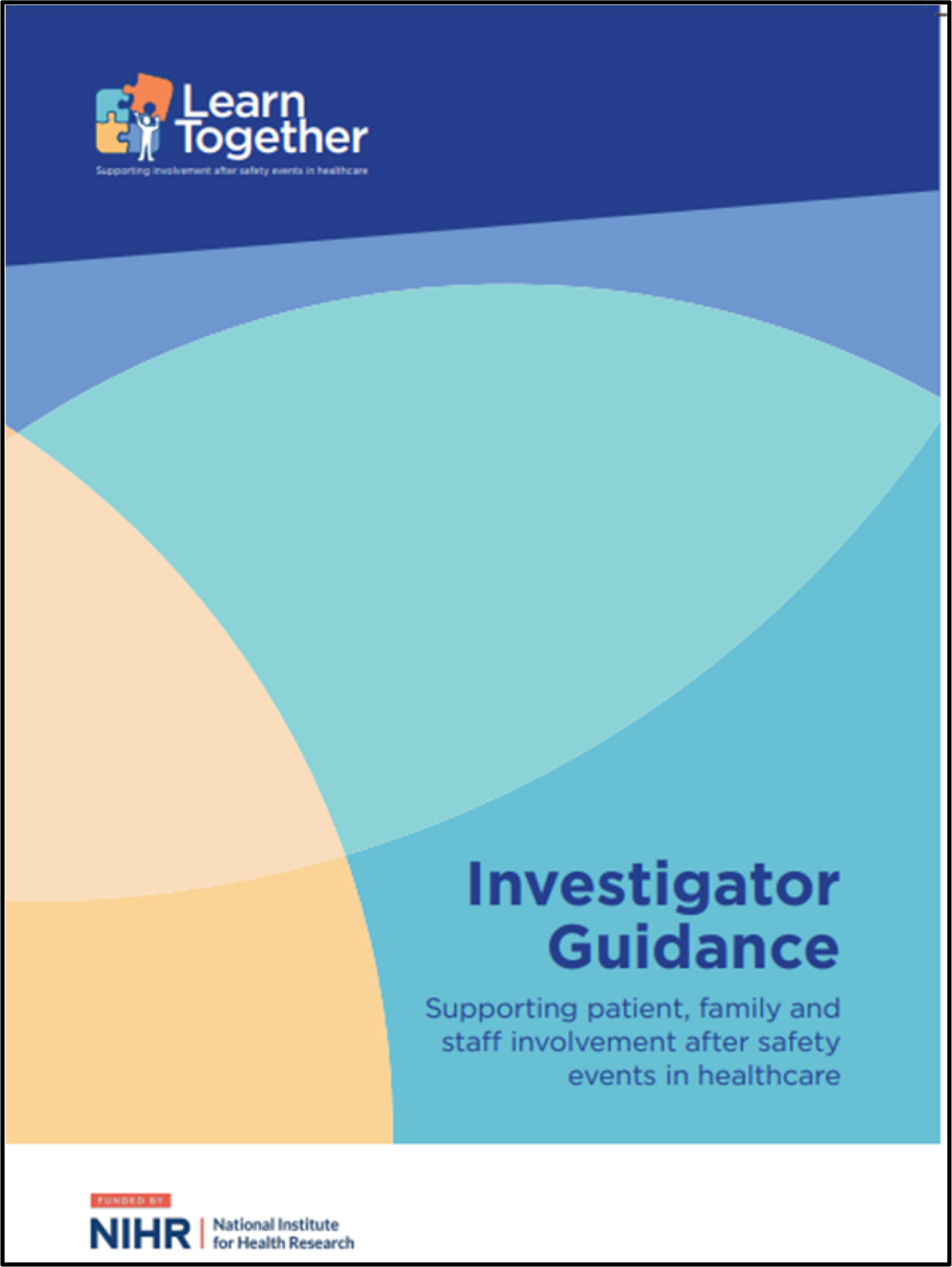

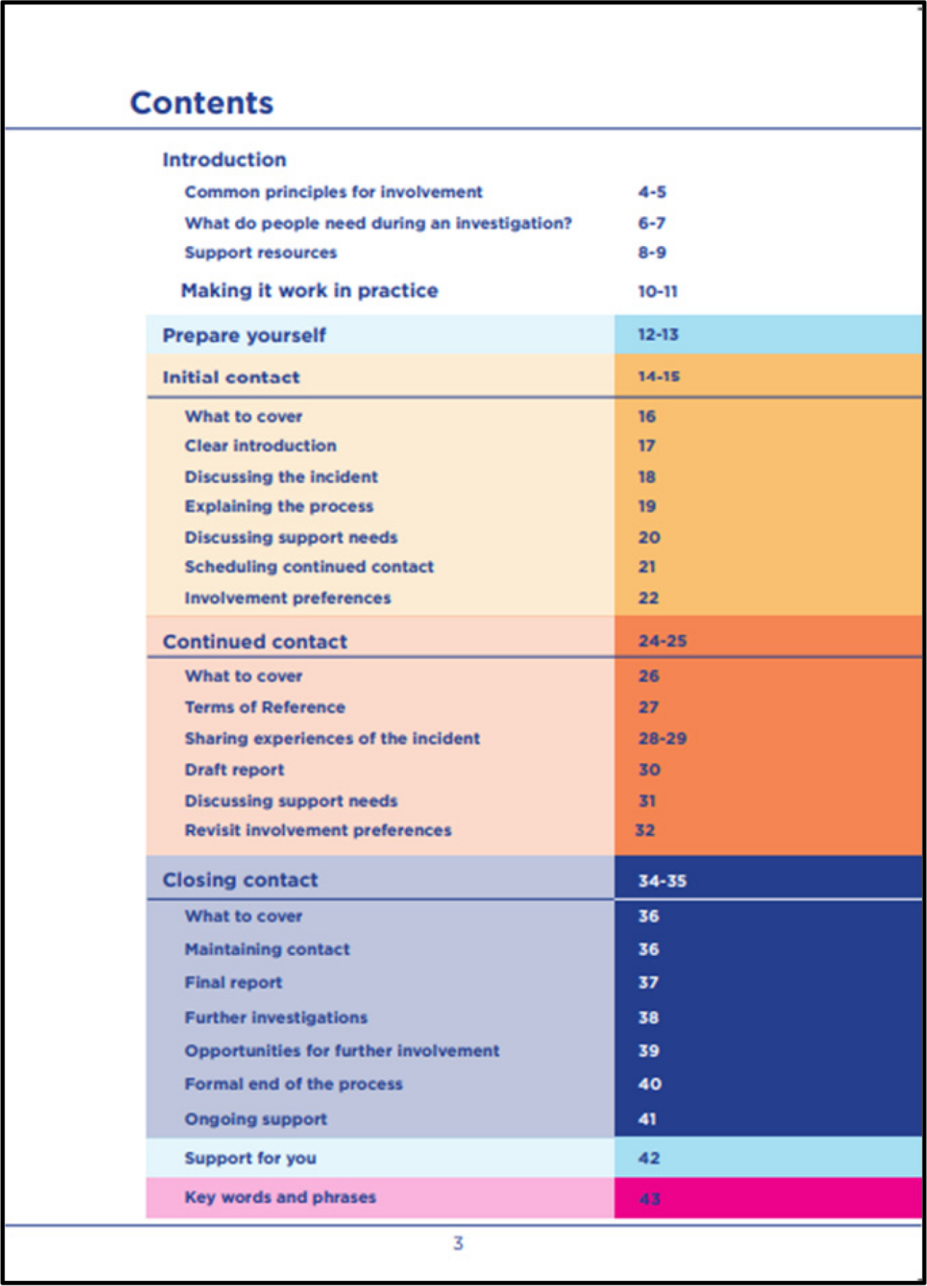
 The Investigator guidance booklet provided an introduction of key background information including the common principles for involvement, what people need during an investigation and sources of support, as well as information about how to make this work for them in practice. Investigators were guided through patient and family involvement in investigations at each stage, broken down into subsections: “prepare yourself”, “initial contact”, “continued contact”, “closing contact” and ’support for you’.

Patient and Family Guidance 
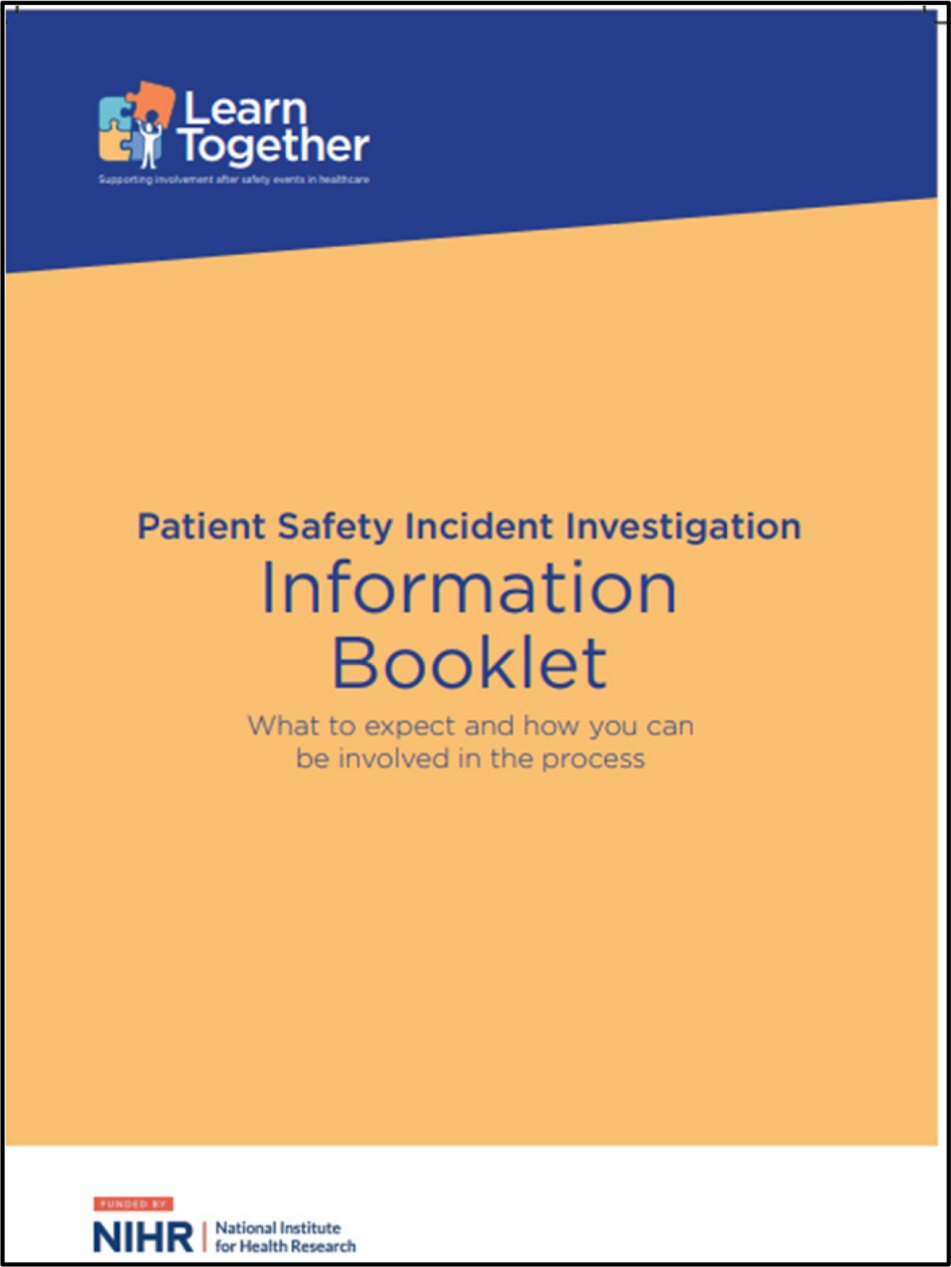

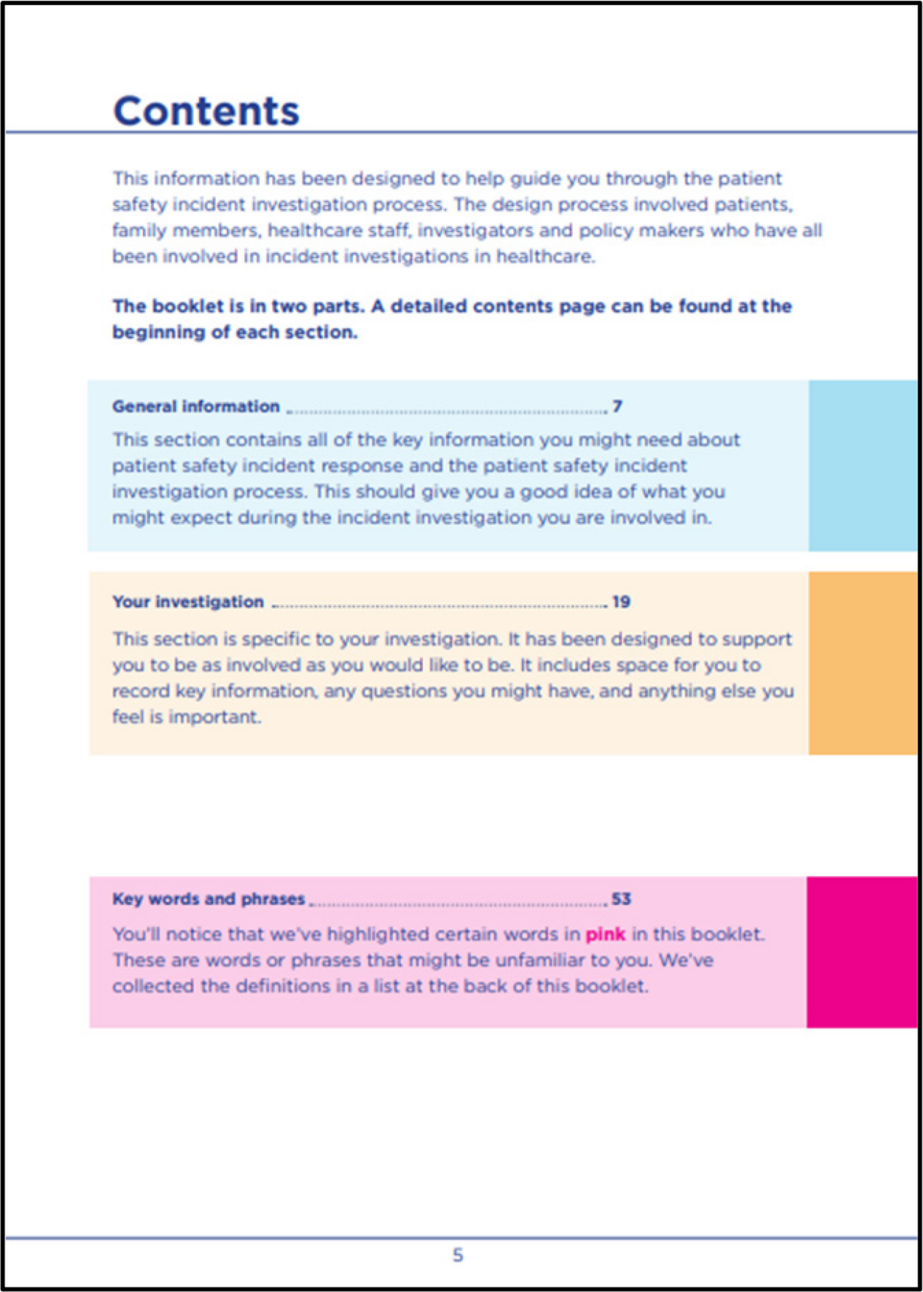
 The patient and family guidance provided information designed to support patients and their families through the process. The content of the booklet was broken down into two key parts. First, “general information”, which was designed to help people know what to expect during the serious incident investigation process. Second, ‘your investigation, which was designed for people to support people to be involved as much as they would like to be, with reflective space to record information, questions and any other information that may feel important.

Other documents:

1. Investigation record

2a. National investigatory body—investigator support

2b. National investigatory body—patient and family reflective booklet

Three other booklets were also developed: (i) an investigatory record, designed to be used by the investigator for individual investigations; (ii) an investigator booklet created for use within the national investigatory body, taking account of the established processes already in place to engage with patients and families; and, (iii) a patient and family reflection booklet to be used to support better engagement with the established national investigatory body processes. The co-designed process and guidance was novel in that while it follows broad chronological steps in an investigation process, it is specifically designed to support relational engagement, with meaningful process engagement being a consequence of successful relational engagement. A real-time evaluation of the guidance, and subsequent iteration (Stage 4 and 5 of the Learn Together programme) is reported elsewhere (41). The iterated versions of the guidance following this evaluation are accessible online (learn-together.org.uk) and are signposted to from the national patient safety policy guiding incident responses within the English NHS (Patient Safety Incident Response Framework, 2022).

## 4 Discussion

Our research aimed to synthesise current evidence to first develop a set of common principles to guide involvement of patients and families in patient safety incident investigations. Using these principles we then sought to develop a working programme theory for how these might be enacted in healthcare organisations. Finally, based on these principles and working programme theory, we aimed to co-design guidance to support the meaningful involvement of patients and families in patient safety incident investigations. We developed ten common principles foundational for the meaningful involvement of patients and families in investigations of healthcare harm, which have utility for any stakeholder in patient safety. Our evidence synthesis found that organizational learning was not the only desired outcome of incident investigations. Importantly, our findings support other work suggesting that when patients and families are not involved in investigations in ways that meet their needs, or where their needs are ignored or minimized, organizations can compound the initial harm (18, 19, 21). Therefore, it is important that investigations seek to reduce the likelihood of any compounded harm being experienced those affected by patient safety incidents. Our programme theory for the co-designed guidance, explicitly features restorative learning at its core; that is, the need to balance organizational learning alongside the minimizing, or eliminating of, compounded harm.

### 4.1 Implications for patient safety investigation theory and practice

Our findings both support and build on existing literature focusing on the application of restorative approaches (22) to patient safety incidents, and a focus on resolution and reconciliation following harm events [e.g., (7, 12–17, 23, 24)].In recent years, interest has been growing internationally in research, policy and practice, in what the foundations are for patient and family involvement in processes that follow incidents, and in particular, incident investigations. For example, Healthcare Improvement Scotland has recently published a set of eight recommendations to underpin better involvement (35), emphasizing open communication, and person-centered approaches, which are consistent with our findings. However, these recommendations were developed through one perspective only—that of patients and families. Our approach here has been to speak to all stakeholders throughout the process of examining the phenomenon, to base our principles in the realities of enacting involvement, whilst meeting the needs of patients and families. An example of this, is the inclusion in our principles of “acknowledge subjectivity”, which recognizes that investigations cannot identify objective truth, but can try to reconcile multiple sources of information, and sometimes conflicting accounts. Such a principle is important if investigators are to avoid raising expectations of patients and families that their information will be prioritized over other sources—an expectation which when not met, might lead to compounding their harm.

By applying and juxtaposing the organizational accident model (20) and restorative approaches (25), we have illuminated new insights, including the important—but sometimes misunderstood—conclusion that organizational learning is not the only, or even the most desired outcome of incident investigations. Our work builds on these existing theories to develop a new “mid-range” theory (36), which we termed “restorative learning”. Our working programme theory effectively provides a set of testable hypotheses about how involving patients and families in investigations in ways that address their multiple needs, might reduce the likelihood of compounded harm and present an opportunity to repair through a collective process of sensemaking. This is an important step towards building testable theory about the experiences and effects of healthcare harm.

### 4.2 Implications for co-design theory and practice

Within our co-design process many of our stakeholders, members of the research team and design team, family members and staff shared reflections that indicated they found the process powerful, enlightening, emotional, transformational, and hopeful. It was a sensitive topic, related to issues of deep, historical harm and trauma, sometimes compounded as repeated poor investigations left staff feeling unsupported, investigators muddling through with insufficient resources, and families left with feelings of injustice, manipulation, dismissal, and duplicity from by the healthcare system. Even within the bounds of the research, it had been a long journey for everyone; for some across decades, often with a sense of activism and ’struggle’ with a political dimension. All this took place in the context of social distancing. Previous in-person co-design experiences suddenly seemed challenging to apply. With such a personal topic, it felt strange to work in seemingly impersonal ways. The art of co-design is about building trust, empathy, and respect between co-design partners even when they have very different, even opposing views, and creating an environment not where people are empowered by us, but where people realize their own power, take their own seat, and speak their own truth. It is also about keeping an eye on the goal of achieving some tangible change by contributing to both macro and micro design decisions (37)—to ultimately make a difference.

Within the co-design and co-production literature, the importance of developing and sustaining good relationships is deemed foundational, but often framed through notions of trust and respect, with relationships left as an implicit but unreferenced concept. Despite these continuing references, there are few academic models or frameworks exploring how to build and sustain good relationships within co-produced research (38, 39).

Understandably, relationships seemed particularly important in this research due to the experiences and trauma many co-design partners had faced. Relationships between the research team and some co-design partners had developed over the course of the research or earlier in developing the proposal. Some partners already knew each other, or of each other. Yet bringing all stakeholders together was an entirely new endeavour. The early phases of the co-design process were carefully developed and delivered to help ease all partners into a “design” frame of mind, emphasising optimism, creativity and empathy for other perspectives. As a team we invested heavily in assessing for and managing pre-existing relationships, as well as developing new relationships, throughout the research process, particularly given the potentially traumatic content and how this might be amplified by coming together with others. This approach has a close alignment with trauma-informed co-design practices that have received much attention since COVID (40, 41). The uniqueness of the Rebuilding Investigations Kit created an important feeling of ’shared’ experience despite everyone doing it alone. The first stakeholder event offered an opportunity to put faces to names and continue the relationship building.

Relationship building was a continual process, across all channels and interactions. The smallest of gestures and phrases accumulated to reinforce that all partners were equal and valued. In other co-design work, we frequently see researchers justifying their decisions to separate patient groups from healthcare professionals, sometimes due to ethical concerns, or on the basis that power differentials might mean people cannot speak freely. We have demonstrated that groups and individuals with very different perspectives and power differences, can be brought together in a process that reduces hierarchy and creates conditions in which all people can speak their truth.

### 4.3 Limitations

The work presented here had a number of limitations. First, the co-design community was a curated group of stakeholders, not research participants, meaning that we did not gather sociodemographic information. Therefore, whilst we went to significant lengths to try to ensure diversity within this group, it may not have been entirely representative of the population of interest. Second, due to the co-design being part of a funded research programme, with specific and predefined aims, resources and capabilities, the collective decisions about what ideas to take forward from the co-design workshops were naturally shaped by these parameters. The other ideas—including for support and advocacy—were valued, but could not be explored further within the confines of the research programme. Further research should explore how, and in what ways, patients and families might be supported through the provision of both organisational, and independent support and advocacy as a mechanism for reducing compounded harm.

### 4.4 Recommendations

This paper has presented the development of principles and co-designed guidance to support patient and family involvement and engagement in patient safety incident investigations. The draft guidance was subject to an extended evaluation, which is described in full in a paper within this research topic (41). However, our findings do support a number of important suggestions for a range of stakeholders.

First, organisations should acknowledge the key role of the investigator, who should be trained and supported by the wider infrastructure to balance the organisational needs with those of patients and families. Any local guidance for investigators or patients and families needs to be flexible and person-centered, and provide navigational support. Importantly, it needs to reflect the relational nature of involvement in investigations and the importance of transparency.

Second, policy makers should be explicit about the dual nature of investigation processes—that organizational learning may not be universally seen as the primary desired outcome, and that this should be balanced with the needs of those involved, and minimizing compounded harm.

Finally, those commissioning and undertaking co-design should recognize that rigour in co-design should not be judged on methodological consistency alone—genuine co-design cannot be achieved by slavishly adhering to a ’standard’ method. However, for those doing co-design, we suggest a focus on building relationships and shifting perspective through the use of creative methods and narratives.

## 5 Conclusions

We have presented a detailed exposition of the important, and often opaque work of evidence synthesis and co-design. This involved, deliberative and extensive process, brought together a multi-disciplinary group of researchers and a heterogenous set of stakeholders, who worked collectively to develop principles and guidance that are grounded in the multiple perspectives and realities of those affected by incident investigations. Our ten common principles and co-designed guidance emphasise two key things. First, that organizational learning is not the only desired outcome for incident investigations, with patients, families and staff reporting the need for restoration and repair. Second, that investigations can be part of reparation, but when it fails to address the needs of stakeholders arising from investigations, it can compound the harm of the original incident. As a result, we have juxtaposed existing theories, and illuminated new insights, proposing a theory of “restorative learning”.

## Data Availability

A minimal data set can be made available by the corresponding author, upon reasonable request.
