# Supplementary material for: The Learn Together programme (part A): co-designing an approach to support patient and family involvement and engagement in patient safety incident investigations
Source: Front Health Serv. 2025 Mar 26;5:1529035. doi: 10.3389/frhs.2025.1529035 (PMC11979208; doi:10.3389/frhs.2025.1529035)
Supplement: Supplementary file 1 [file Datasheet1.pdf]

PFI-SII
Involving Patients and Families in Serious Incident Investigations

Hello, it's nice to meet you.

If you would like a member of the team to help you read or complete this document, or to answer any questions, please get in touch using the following details:

For questions on the design process and your involvement, contact Rebecca at **r.partridge@shu.ac.uk**

For questions about the wider project or to discuss any concerns, contact Lauren at **l.ramsey@leeds.ac.uk** or Siobhan at **siobhan.mchugh@bthft.nhs.uk**

What's in the Box?

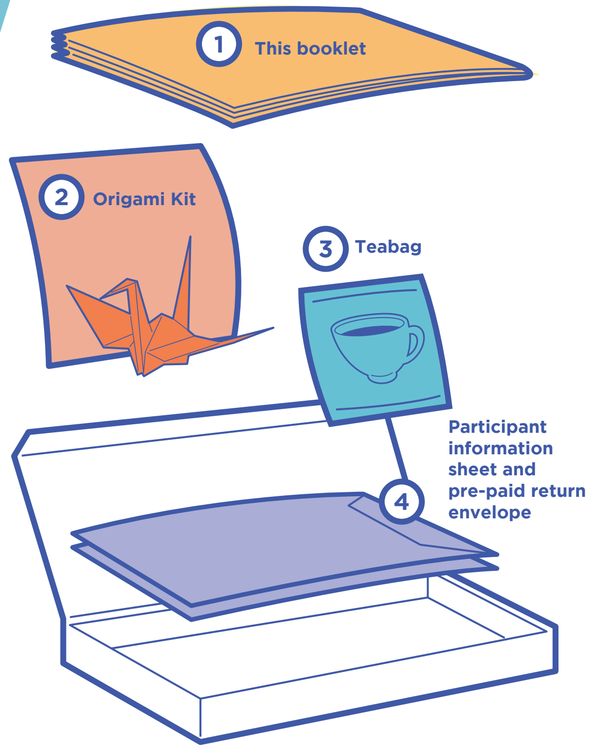

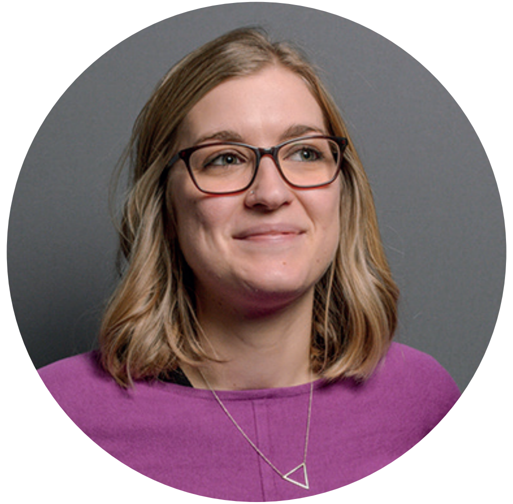

"Hello, welcome to the start of the design phase of the Serious Incident Investigations Co-design Project. I'm Rebecca, one of the Design Researchers who will be leading this phase. You can find out more about our team on page 11.

Receiving this pack marks the beginning of this stage of the project. Please keep this booklet safe, it contains important information about the project, co-design sessions and what to expect. You can come back to it at anytime and our contact details, should you need them, are on the opposite page.

Thank you for joining us, we know how precious your time is and it's great to have you on board."

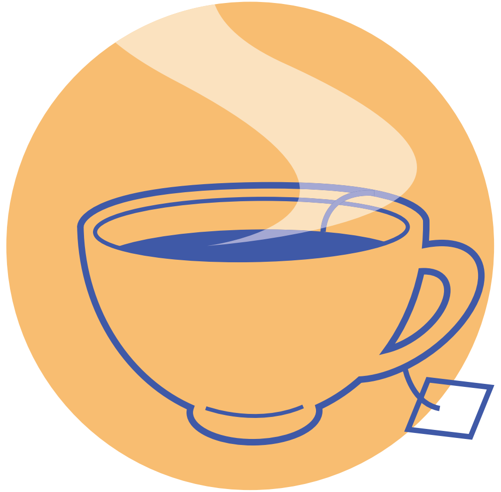

All the best things start with a hot drink. You'll find a teabag included in your box, so take a moment to put the kettle on and have a brew while we tell you a bit more about the project.

In this booklet.

Project Information.
Overview and Team members

Lab4Living.
Co-design

What to expect.

Your Information.
Next Steps

Project Information.

It is estimated that ten thousand cases involving harm or death are reported in the NHS every year. Some of these cases are investigated as serious incidents by the NHS. **Indications suggest that greater involvement of patients and families in these investigations leads to better learning from such incidents, meaning they are less likely to occur in the future.** However, there is currently limited UK evidence to guide organisations in meaningfully involving patients and families in serious incident investigations.

What we hope to achieve.
This project aims to develop guidance to support more **meaningful involvement** of patients and families in serious incident investigations.

How we hope to achieve it.
We are working collaboratively with people who have experience of serious incident investigations (that's you) via a process called co-design. We will then test this new guidance out in live investigations.

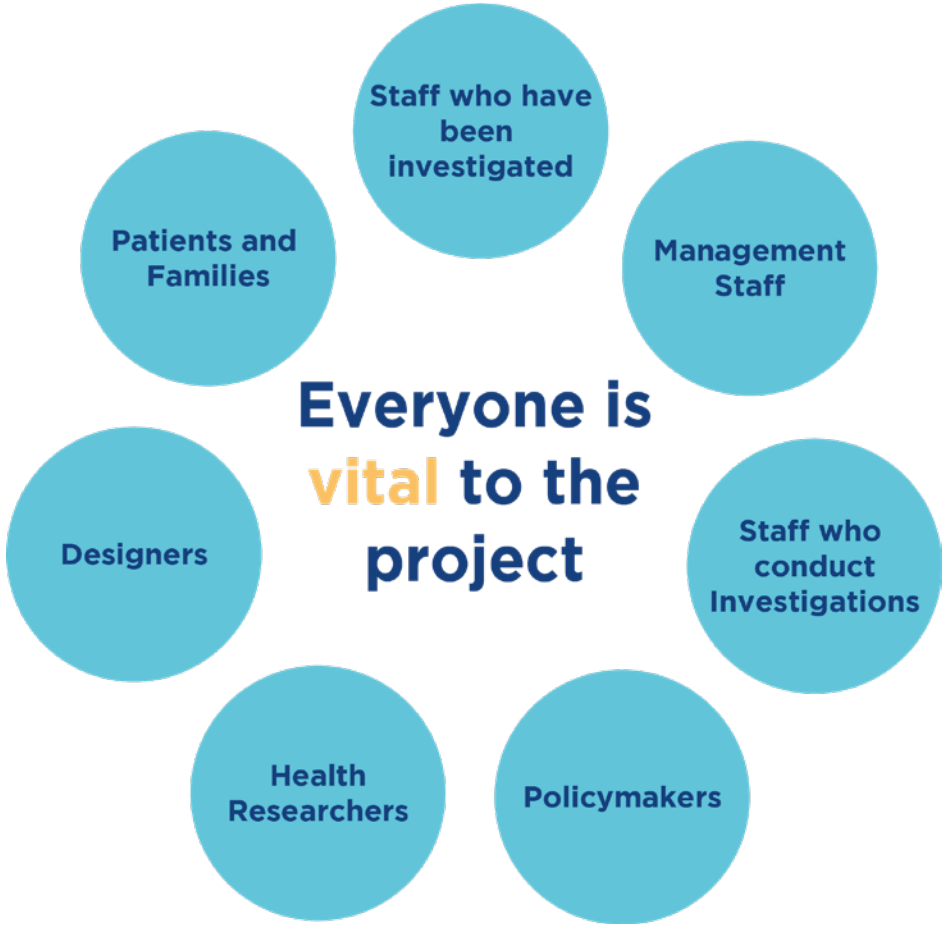

Project Overview.

This project spans three years. The bit that you're involved in now is the second year. There are a range of activities happening across this year, as you can see on page 17.

At the end of year three we'd like to invite you back to an event to celebrate everyone's contributions and share the outcomes of the project. We'll be in touch with a date closer to the time.

We're here!

Final event

Year one

Interviews

Documentary Analysis of policies

Literature Review

In the first year the research team interviewed people who have experience of serious incident investigations. They looked at current policy documents along with published literature to understand current experiences.

Year Two

Co-Design Activities

The focus of the second year is to take the learning from year one and over a series of interactions work together to design new processes to support more meaningful involvement of patients and families in serious investigations.

See page 17 for a detailed view of what we have planned in year two.

Year Three

Trial of new processes

During the final year, the new guidance will be applied to live investigations. The team will talk to those involved in these investigations to understand their experiences. This will help us to evaluate the guidance.

Team Members.

This project draws on expertise from multiple areas. You may have already met the research team from Bradford. If not, then here are some of them who you might meet during your involvement in this project.

Whilst this team are overseeing the whole project and have conducted all the activities during the first year it is the team at Lab4Living who will be running the design phase discussed in this booklet. You'll find out more about them on the next page.

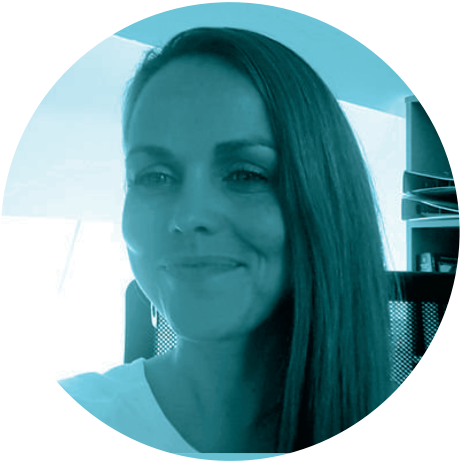

Jane is a Professor of Healthcare Quality & Safety. Her expertise is in patient safety and she leads this research project.

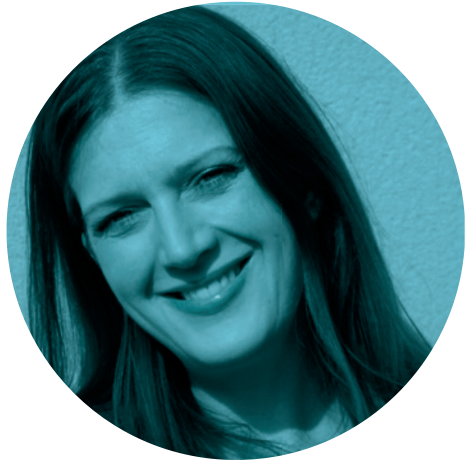

Ruth is the PFI-SII Programme Manager. Her expertise is in psychological trauma, mental health and wellbeing.

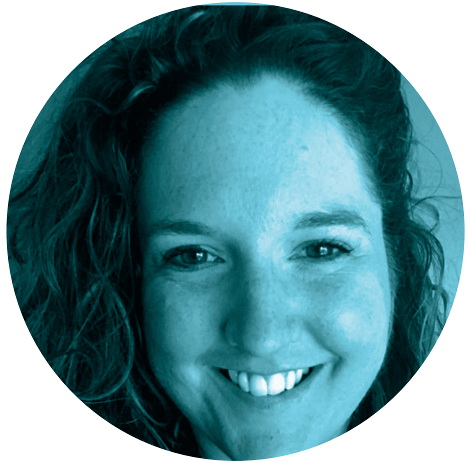

Siobhan is a Research Fellow. Her expertise is involving patients, families and staff in health service improvement and patient safety.

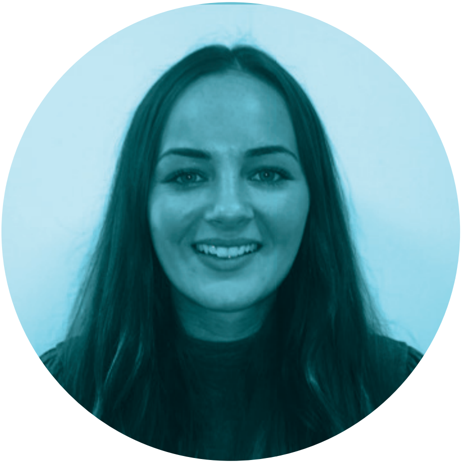

Lauren is a Research Fellow. Her expertise is in exploring different perspectives of patient and family involvement in patient safety.

Lab4Living.

Lab4Living is a collaborative community of researchers in design, healthcare and creative practices. Our work spans more than 100 research projects and has included collaborations in over 80 academic, hospital and community organisations in over fifteen countries.

Here at Lab4Living our expertise is in designing things with end users like yourselves. It's important to know that we're experts in co-design but not serious incident investigations. As far as we're concerned, if you are reading this then you are the expert. If you've never been involved in co-design before there's more information on the next page.

Becca is a Design Researcher. Her expertise is in the facilitation of creative methods.

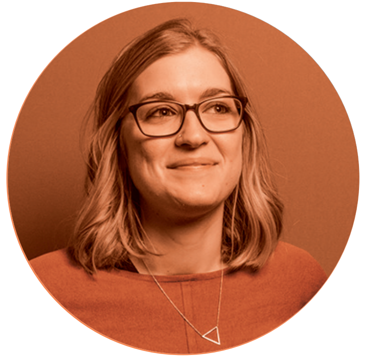

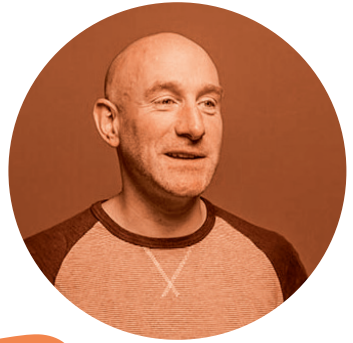

Joe is a Principal Research Fellow. His expertise is knowledge mobilisation.

Chris is a Designer and Illustrator. His expertise is making knowledge accessible to everyone.

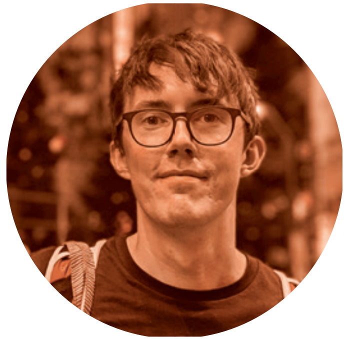

- LAB4LIVING - EXCELLENCE IN COLLABORATIVE DESIGN RESEARCH

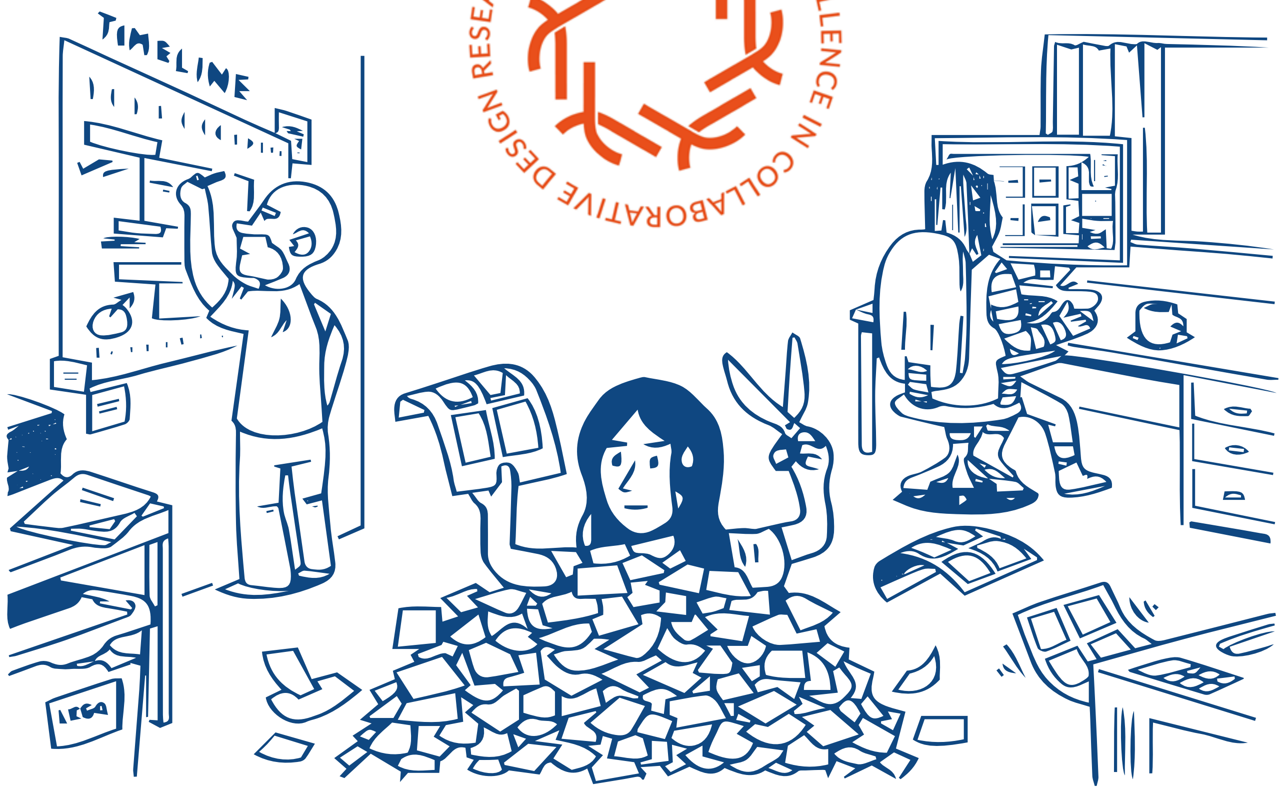

Co-design.

Co-design describes a design process used in research where **designers and those with experience or knowledge of the thing to be designed, work together.**

Co-design spans the whole design process from **understanding experiences, deciding design priorities, proposing solutions, and developing the desired outcome.** It doesn't dwell on the past, but looks to create new and better futures.

In co-design, designers, other researchers and co-design partners (you) share knowledge, skills and experiences. **It is creative and uses a range of methods.**

You will be asked to draw upon your previous experiences to create these better futures - but we won't dwell on the individual. Therefore **there may not be an opportunity for you to share your personal experience completely.**

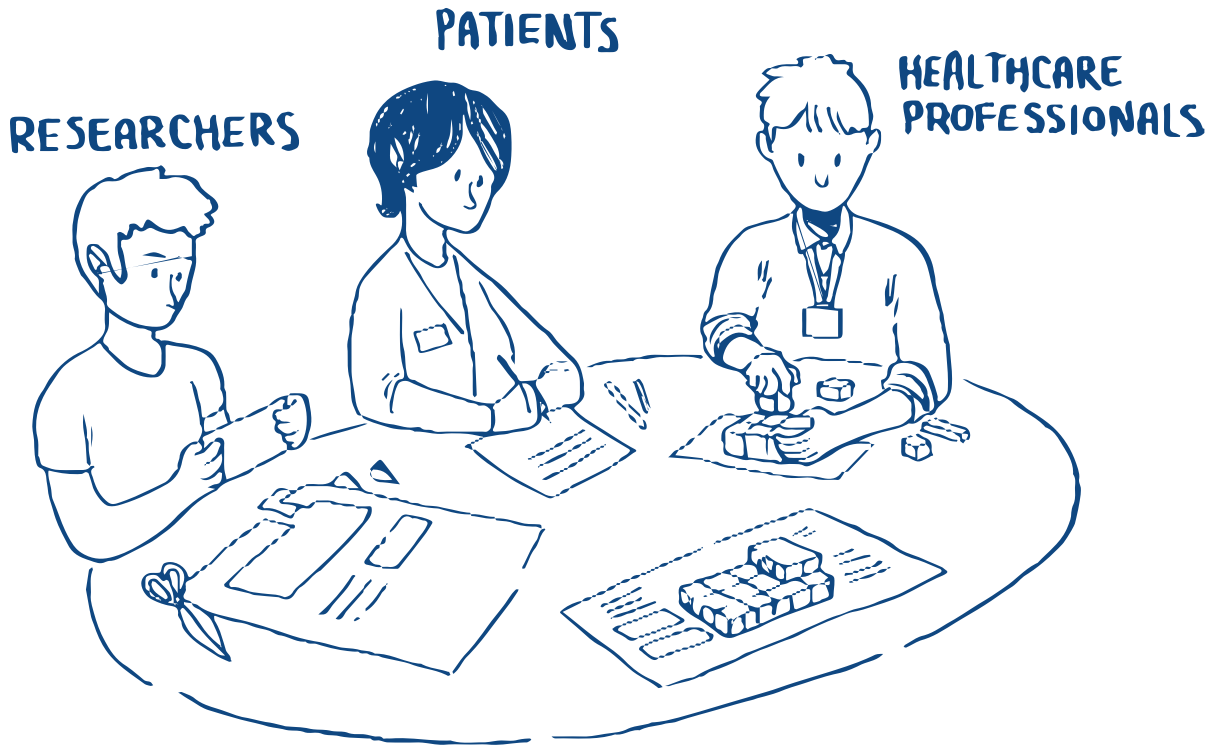

Co-design.

The majority of our co-design work is usually done through face-to-face in workshops. We've had to change this because of Covid restrictions, and now use a much wider range of methods (like this one). We're learning so much as we do this and would appreciate any feedback you have throughout the project.

One of the key tenets of co-design is creative activities and learning through doing or making.

Throughout this process you'll be involved in a range of creative activities. Through these we'll invite you to 'do' something, to make, create or respond to something through more than just words. We find this helps people to access a deeper level of thoughts, feelings, information and ideas.

To get you started on this journey we'd like to invite you to take part in a small making activity. In this box you'll find a simple origami kit. You'll find further information and instructions in the kit.

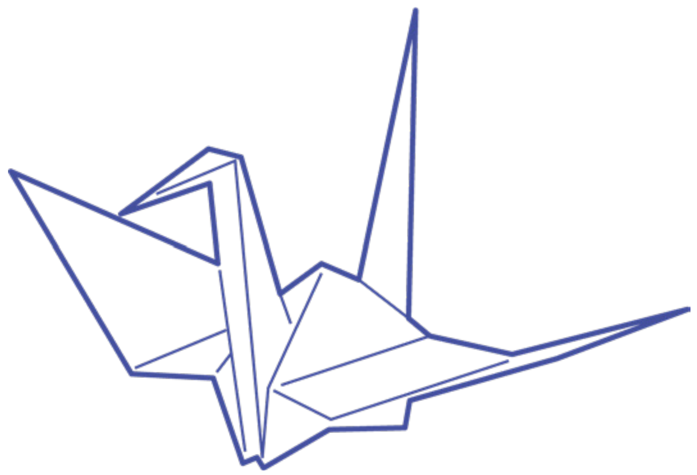

What to expect.

Here's an overview of what your involvement will look like. There are around 50 people involved in the project in total, who will all meet for the stakeholder events. For the co-design sessions you'll meet in smaller groups of around 15, these are specific to a workstream; acute, mental health or national investigations.

We are asking that everyone attends all the events they have signed up for. We are trying to accommodate ways that people can maximise their project involvement. We'll ask you for more information on this in information sheet.

Co-Design workshop dates

Mental health: 17th May & 5th July
Acute Health: 19th May & 7th July
National Level: 21st May & 9th July

All workshops are from 1-4pm and will be held virtually using zoom. You will receive a link nearer the time.

Year Two

- Invited to co-design
- Introductory Pack — We're here!
- Investigating the Investigation — **Arriving on your doorstep w/c 12th April:** An at-home activity to complete. You will be invited to reflect on an example serious incident investigation and the different perspectives throughout.
- Stakeholder Event — **21st April:** A full project event to establish common principles based on the findings from year one and reflection on the building stories kits.
- Co-Design Workshop One / Co-Design Workshop Two — Smaller workstream groups — **W/c 17th May & 5th July (see box on left for workstream dates) :** Using the common principles we'll consider what the new processes might look like and refine them to make sure they work for everyone.
- Stakeholder Sharing Event — **22nd September:** Final sharing event to reflect on what has been learned, and agree the final processes and guidance

Year Three

- **Date TBC:** A full project celebration event.

Your Information.

Throughout this process we'll send things to you and ask you to send things back to us in return (electronic or via post). As we're all doing this research from our own homes, these are the best ways for us to share information.

The first thing to send to us is some information and your communication preferences. This helps us to get to know you a bit better and ensures we have all we need to support you.

You may have already provided some of this information to the research team, but this ensures that the Design Researchers are communicating in the way that best suits you.

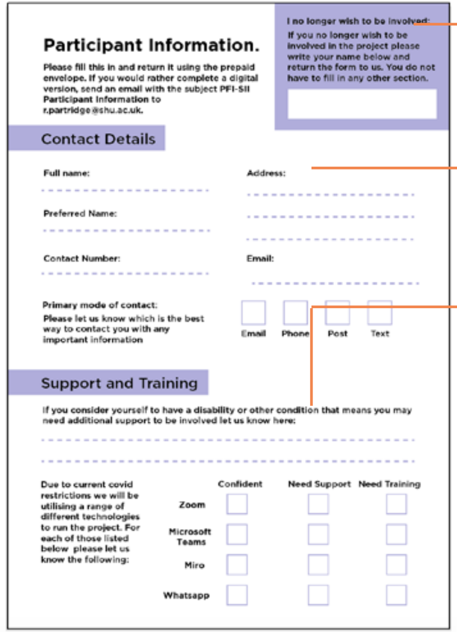

Participant Information.

Please fill this in and return it using the prepaid envelope. If you would rather complete a digital version, send an email with the subject PFI-SII Participant Information to r.partridge@shu.ac.uk.

I no longer wish to be involved:

If you no longer wish to be involved in the project please write your name below and return the form to us. You do not have to fill in any other section.

Contact Details

Full name:

Address:

Preferred Name:

Contact Number:

Email:

Primary mode of contact:
Please let us know which is the best way to contact you with any important information

Email Phone Post Text

Support and Training

If you consider yourself to have a disability or other condition that means you may need additional support to be involved let us know here:

Due to current covid restrictions we will be utilising a range of different technologies to run the project. For each of those listed below please let us know the following:

	Confident	Need Support	Need Training
Zoom			
Microsoft Teams			
Miro			
Whatsapp			

If you would rather return this information electronically please send an email with the subject heading **PFI-SII participant information** to r.partridge@shu.ac.uk. I will send you a digital version of the form.

If you no longer wish to be involved in this project, fill in this section only.

Contact Details: So we know how best to contact you.

Support & Training: To make it as easy as possible for you to be involved or you require additional support or training needs.

Information about you: So we can get to know who we're working with.

Payment: If you are not participating in your professional capacity, or are participating during your own time (on leave, days off etc.) then we are happy to pay you for your time, at rates suggested by INVOLVE. Please tick here if you want us to send information to you to claim for your time in this co-design community.

Experience: So we can understand the range of experience within the group.

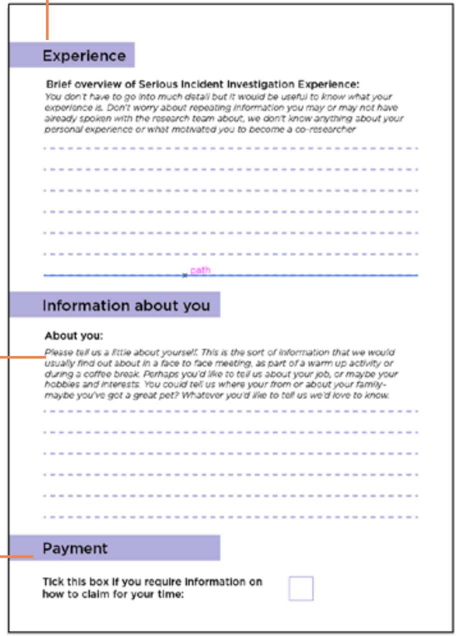

Experience

Brief overview of Serious Incident Investigation Experience:
You don't have to go into much detail but it would be useful to know what your experience is. Don't worry about repeating information you may or may not have already spoken with the research team about, we don't know anything about your personal experience or what motivated you to become a co-researcher

path

Information about you

About you:
Please tell us a little about yourself. This is the sort of information that we would usually find out about in a face to face meeting, as part of a warm up activity or during a coffee break. Perhaps you'd like to tell us about your job, or maybe your hobbies and interests. You could tell us where your from or about your family- maybe you've got a great pet? Whatever you'd like to tell us we'd love to know.

Payment

Tick this box if you require information on how to claim for your time:

What's next?

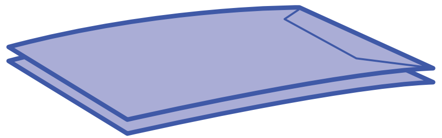

Put your Information sheet in the post. Unless you say otherwise, we'll assume you still want to be involved. **If we do not hear from you by April 19th then the design team will follow up to confirm your information and preferences.**

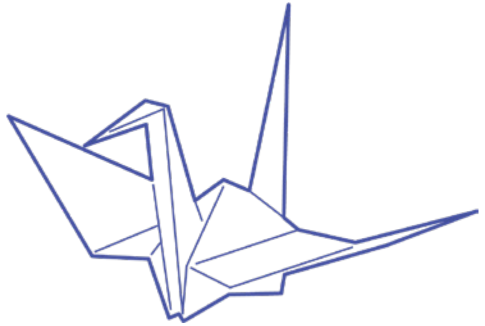

Have a go at your origami kit.

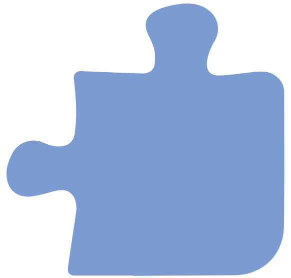

Keep an eye out for your Investigating the Investigation kit. This will arrive on or close to the 12th April. Please complete this before the first stakeholder event.

Have a great rest of your day.

SUPPORTED BY

NIHR | National Institute for Health Research

PFI-SII

Involving Patients and Families in Serious Incident Investigations
