# Supplementary material for: The Learn Together programme (part A): co-designing an approach to support patient and family involvement and engagement in patient safety incident investigations
Source: Front Health Serv. 2025 Mar 26;5:1529035. doi: 10.3389/frhs.2025.1529035 (PMC11979208; doi:10.3389/frhs.2025.1529035)
Supplement: Supplementary file 2 [file Datasheet2.pdf]

Learn Together

Supporting involvement after safety events in healthcare

Investigator Guidance

Supporting patient, family and staff involvement after safety events in healthcare

FUNDED BY
NIHR | National Institute for Health Research

Introduction

This Investigator Guidance has been specifically designed to support you to involve patients, family members and healthcare staff in patient safety incident investigations.

The Guidance aims to give you practical ideas about how to support involvement throughout an investigation, as well as providing you with information about why involvement of different stakeholders is important. We know that each investigation you undertake will be unique, and that different people will want different levels of involvement which might change over time. We also understand that you will have your own way of working during an investigation. But we hope this Guidance provides you with flexible ideas to support different individual needs, embedded within your own ways of working and your organisational investigation policies.

"Every investigator that you meet along the way, tell them how important they are; how valuable they are to ensure that people don't walk the route that [we] have had to walk. They are vital to systems and should be valued at every single step, because the difference they make is immeasurable."

Debra Hazeldine MBE.

Debra's Mum Ellen died in Mid Staffordshire.

© Copyright Bradford Teaching Hospitals NHS Foundation Trust, 2021. All rights reserved. This material must not be reproduced or shared without permission from Professor Jane O'Hara (jane.o'hara@bthft.nhs.uk).

Contents

**Introduction**	
Common principles for involvement	4-5
What do people need during an investigation?	6-7
Support resources	8-9
**Making it work in practice**	10-11
**Prepare yourself**	12-13
**Initial contact**	14-15
What to cover	16
Clear introduction	17
Discussing the incident	18
Explaining the process	19
Discussing support needs	20
Scheduling continued contact	21
Involvement preferences	22
**Continued contact**	24-25
What to cover	26
Terms of Reference	27
Sharing experiences of the incident	28-29
Draft report	30
Discussing support needs	31
Revisit involvement preferences	32
**Closing contact**	34-35
What to cover	36
Maintaining contact	36
Final report	37
Further investigations	38
Opportunities for further involvement	39
Formal end of the process	40
Ongoing support	41
Support for you	42
Key words and phrases	43

Common principles for involvement

Every investigation will be different, due to the range of incidents that can occur and the different levels of involvement that people might want or need. It is difficult to standardise your ways of working during an investigation because of this. You need to maintain as much flexibility as possible, especially when thinking about involvement. We have developed a set of **common principles for involvement** that can be flexibly applied in context.

The common principles were drafted following a series of interviews with patients, family members, healthcare staff and investigators who all have experience of the serious incident investigation process. Through their reflections on their personal, very varied, experiences we were able to draft a set of principles that support meaningful engagement with, and involvement of, those who have experienced the incident.

There is space beside each principle for you to capture any general reflections you might have about them.

There will be an opportunity to think about how you might apply these principles during an investigation in the section '**Making it work in practice'** from **page 10** onward.

Make apologies meaningful. Rather than offer excuses, demonstrate understanding and a commitment to learn what has happened and why.

....
....
....

Individualise your approach. Involvement should be flexible and adapt to changing needs. Set realistic expectations.

....
....
....

Be sensitive to timing. Investigations can feel like they're happening slowly, quickly or at insensitive times. Investigators need to manage time carefully.

....
....
....

Treat people with respect and compassion. Harm can happen through the experience of the investigation, and how people are treated within it.

....
....
....

Strive for equity. Investigations allow an organisation to learn, but if their agenda is prioritised over patients/families/staff, the process can feel discriminatory.

....
....
....

Provide guidance and clarity. Patients, families and healthcare staff can all be confused by what an investigation actually entails.

....
....
....

Listen. If there is a true commitment to learning, then everyone involved should have the opportunity to share their experience.

....
....
....

Be collaborative and open. People who feel involved are less likely to need to seek other routes to be heard (e.g. complaints, litigation).

....
....
....

Respect humanity. Investigations should embrace and accommodate different human responses.

....
....
....

Accept subjectivity. Each individual will experience the same incident in different ways. No one truth should be prioritised over others.

....
....
....

What do people need during an investigation?

An investigation is like a jigsaw. Everyone involved will bring different information about the incident.

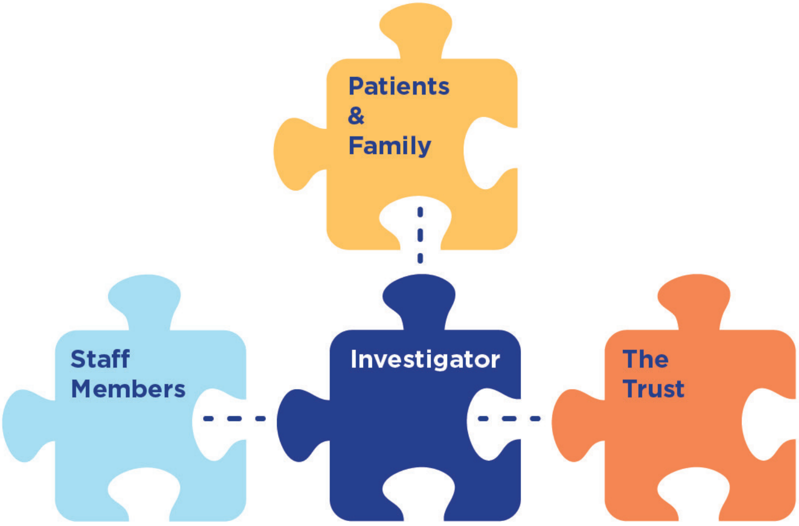

Everyone involved in the investigation will have needs that might fluctuate and change as it progresses. These needs could be *emotional, practical or psychological*. We understand it's not your role to address every stakeholder's needs. However, it is important to be aware of these needs so that you can support people to access additional help where required.

Following a series of interviews with patients, family members, healthcare staff and investigators who have all experienced serious incident investigations, we were able to understand more about the most common needs for different groups and how they changed through the investigation process. We know an investigation is unlikely to be a linear process, but we have tried to identify needs linked to some of the key parts of an investigation.

These **common needs** are just a guide. Involving people during the investigation process means you are more likely to understand their individual needs. Don't worry if you aren't able to attend to these needs in your role as an investigator. Understanding what people need means you can signpost them to other appropriate services if necessary.

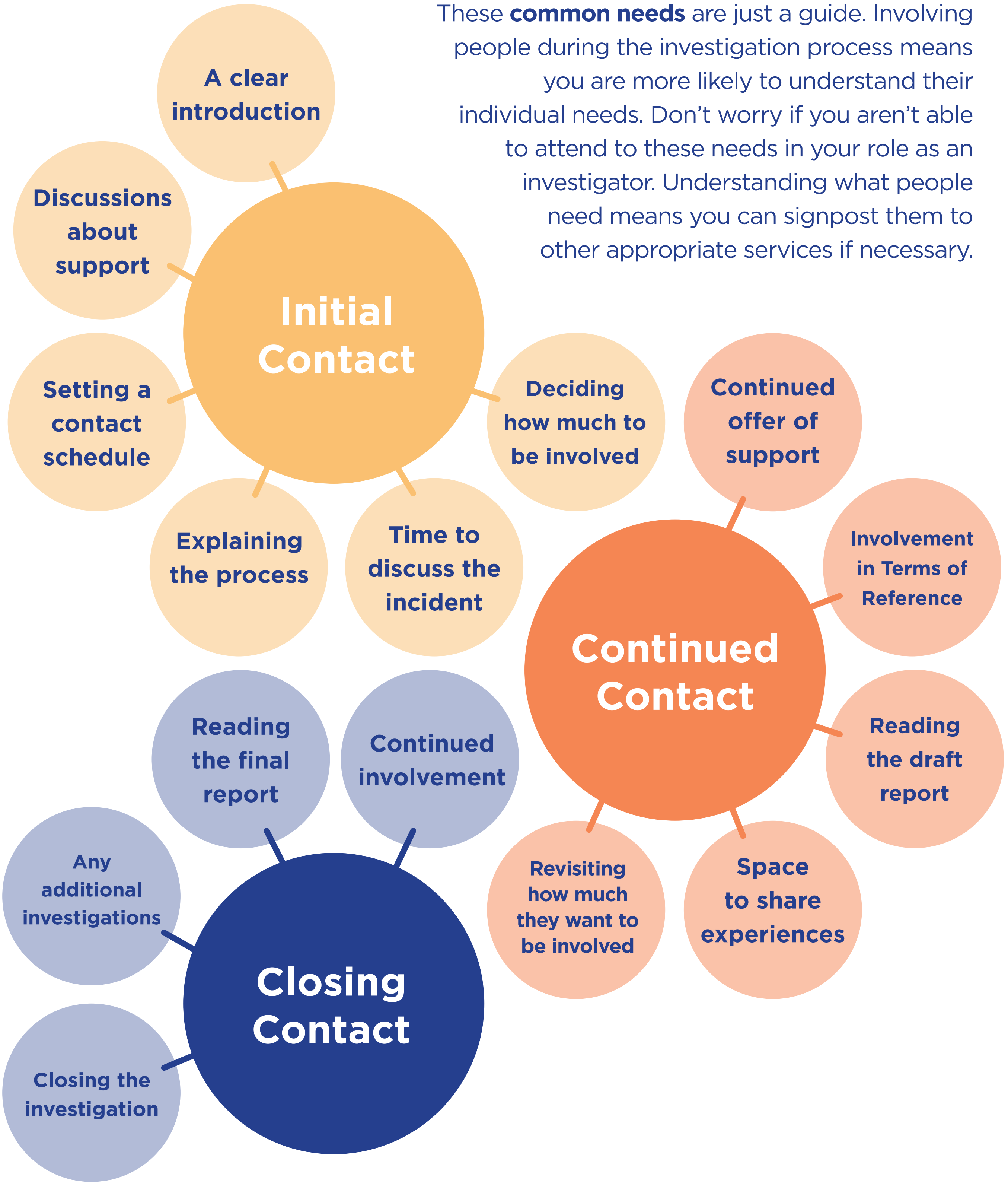

Support resources

This guidance is part of a suite of resources designed to support you to more meaningfully involve patients, families and healthcare staff in patient safety incident investigations. The additional resources available to you are:

Learn Together Information Booklets

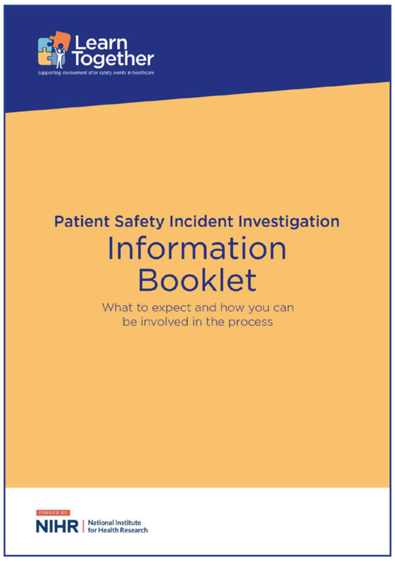

Patient & Family version

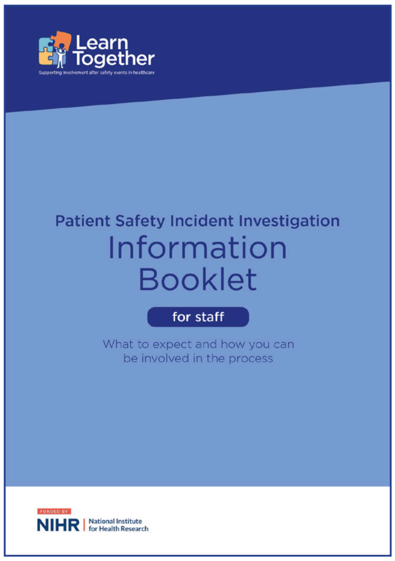

Staff version

The information booklets have been designed to support patients, family members and healthcare staff through a patient safety incident investigation. The booklets are set out in two parts:

- The first part provides general information about patient safety incidents and patient safety incident investigations. This will ensure everyone involved in the investigation has a good idea about what this process involves.

- The second part provides patients, family members and healthcare staff space to record important details about their specific investigation. It also signposts them to more general support organisations.

Making it work in practice

This section we will provide you with practical ideas about how to support meaningful involvement of patients, family members and healthcare staff during an investigation. We will break down the key points of communication, and ground the practical ideas in the common principles for meaningful involvement.

We hope that this guidance will not only provide you with ideas about **how** to involve different people in investigations, but also the reasons **why** this is important.

Preparing yourself

It is important to be aware of the experience you bring and to prepare accordingly. Patients, family members and staff members are also likely to be more prepared for the investigation after receiving their information booklets.

You might want to:

- ☐ Read through the information booklet that patients, family members and staff will receive.
- ☐ Have a copy of your **Trust policy** about patient safety incident investigations to hand.
- ☐ Familiarise yourself with the current **NHSEI policy** on patient safety incident responses.
- ☐ Explore information about **additional investigation processes** if appropriate (e.g. coroner, complaints etc).
- ☐ Explore incident specific **support resources** nationally and locally where appropriate.
- ☐ Consider the **terminology** used throughout an investigation.

Specialist terminology

You can find a glossary of key words and phrases (highlighted throughout all resources in **pink**) on **pages 43-45** of this guidance booklet. Importantly, **this is the same glossary provided to patients, family members and healthcare staff in their information booklets**. This means you can see what they might understand by certain terminology. You can also refer them to it if necessary.

Support resources

The information booklets all include contact details for organisations that can provide general emotional and practical support. It might also be helpful to search for more specific support organisations, taking into account the incident, the circumstances of the people involved, and other social, practical or cultural needs people might have.

Policy documents: Trust-level and national policies

In the information booklets we advise patients, family members and healthcare staff that you will use the **Trust investigation policy** as a guide during the investigation process. They might ask to see a copy of this policy document, or ask you questions about it. You might want to have a copy of the Trust policy document to hand during the investigation.

Patients, family members and staff are also directed to the **NHSEI Patient Safety Incident Response Framework (PSIRF)** via the NHS website https://www.england.nhs.uk/patient-safety/incident-response-framework/. They might ask you to direct them to this, or ask you questions about it and how the national and local documents differ. You might want to familiarise yourself with the general purpose of the national document before the investigation.

Additional investigations

Depending on the incident there may be additional investigation processes happening concurrently with, or following, the Trust level investigation such as a **coroner's** inquest or an investigation by the **Healthcare Safety Investigation Branch (HSIB)**. If you suspect that additional investigations are likely, you should prepare to discuss this with the patients, family members or healthcare staff involved in the investigation. You can find out more about inquests at https://resolution.nhs.uk/wp-content/uploads/2020/03/Inquests-films-and-guide.pdf and about the HSIB here https://www.hsib.org.uk/.

The patient safety incident investigation might also have been triggered by the complaints process, rather than standard internal processes. If this is the case, you should be prepared to explain to the people involved why the Trust didn't initially plan to investigate this incident as a PSII, and answer any questions they have. You can find out more about the NHS complaints process at https://www.nhs.uk/using-the-nhs/about-the-nhs/how-to-complain-to-the-nhs/.

Initial contact

Ideally, you will make first contact with the patient or family member, and the healthcare staff involved, at the beginning of the investigation. However, we know that this might not always be possible. Although the timing of making this first contact might be flexible, the principles of making the first contact with people who have experienced the incident are important to get right. Initial conversations with anyone who has experienced the incident will set the tone for the rest of the investigation.

Patients and family members will have had varied experiences of healthcare provision leading up to the incident, and different experiences of the **Duty of Candour** process. They might be recovering physically, psychologically or emotionally from the incident, or the impact of it. **Healthcare staff** will respond to involvement in an incident differently, and will have received varied levels of support afterwards.

You are not expected to know in detail their experiences up to this point, but your role at this point is to set a tone you are both comfortable with moving forward. Enacting the **common principles for involvement** is likely to make the experience of this initial communication less daunting, and more meaningful, for you and them:

Respect humanity

Different people will respond differently to the same incident. Use your initial conversations to determine the impact of the incident on each individual you involve. This will make it easier for you to set an appropriate tone with each person and to accommodate their involvement needs.

Treat people with compassion

Harm can be caused during the investigation and the way that people are treated within it. By being respectful, compassionate and empathic in these early conversations you can prevent this additional harm.

Strive for equity

Organisational learning is the main output of an investigation, but meaningful involvement of the people who experienced the incident can augment learning whilst making the process feel less discriminatory.

Make apologies meaningful

In your initial conversations you should give a meaningful apology for the incident where appropriate, and demonstrate your commitment to learning about what happened.

Patients, family members and healthcare staff will know less than you think about the incident and the investigation process. The period after an incident can be overwhelming. **Patients and family members** will have already been through Duty of Candour and might have received communication about the investigation. They might also be receiving continued healthcare, or have additional things to attend to such as home adaptations, additional care arrangements or funeral planning. **Healthcare staff** will have been involved in reporting the incident, and might have been involved in preliminary investigation processes such as a 72 hour review or a panel meeting. You should use your initial conversations with people to check they understand what the incident was and why it is being investigated.

Provide guidance & clarity

You should be clear in your initial communications about who you are, what has happened and what the investigation might entail. It can be a confusing to take in and process a lot of different information, especially at what might be a difficult or emotional time.

Listen

The time following an incident might be particularly emotional for the people who experienced it. People might come into the initial conversations with you with things they want to tell you or questions they want to ask you. Listening to them will help you check their understanding and will demonstrate to them your commitment to learning and to involvement.

On the following pages you will find ideas about how to make the most of your initial contact and conversations.

What to cover

The following checklist should give you practical ideas about what you should include in your initial conversations with patients, family members and healthcare staff after an incident. You don't have to do everything in one conversation, but these are things you should aim to cover in the initial period of involvement. You might find you do a lot of these things already.

- ☐ Introduce yourself clearly as the investigator.
- ☐ Explain what the incident was and check their understanding.
- ☐ Explain what will happen during the investigation.
- ☐ Ask if they have any questions about the investigation process.
- ☐ Discuss any potential support needs.
- ☐ Ask how they would like to be contacted and how often.
- ☐ Check whether there are any specific dates they do not want to be contacted.
- ☐ Discuss their preferences for involvement.

Clear introduction

You should introduce yourself clearly to anyone you involve in the investigation. To maintain equity of access to involvement in the investigation patients, family members and healthcare staff should be able to contact you as well as you being able to contact them.

- **Give the patient or family member your full name.**
  Check if they would like you to repeat it.

- **Tell them you are the lead investigator and where you work.**
  This might be the specific hospital name or the name of the Trust.

- **Tell them if you also have another role within the Trust or externally.**
  You should make them aware if you are not a full time investigator as this will mean their expectations of your time will be different.

- **Give them your contact details and discuss your working hours.**
  You should make it clear how and when they should contact you and set clear expectations about when they should expect you to reply.

Can you have this conversation over the telephone, video call or face-to-face to make it more personal?

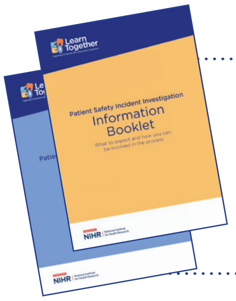

The information booklets have space on **page 23** for people to record these details. You could use these pages to structure your introductory conversations with patients, family and staff.

Discussing the incident

Explain what the incident was, clearly and in language appropriate to the person you are talking to. Try and explain to the person why the decision was made to investigate the incident, if you know. Once you have provided a clear explanation of the incident, you should check the person has understood.

- **Tell them what you are investigating.**
  Give them a brief explanation of the incident and why the decision has been made to investigate it.

- **Check their understanding.** Ask them appropriate questions to check they have understood what you have told them about the incident and why it is to be investigated.

- **Direct them to their information booklet for more general information.**

Could you have this conversation on a video call or face-to-face to make it easier to check their understanding, and respond to them more appropriately?

Could you offer people the opportunity to have someone with them while you have this conversation? They might be more likely to take in the details of the incident.

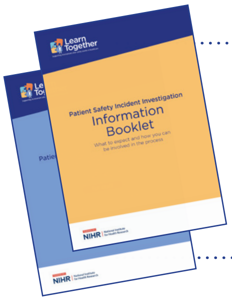

The information booklets have space on **pages 24-25** for people to record details about the incident. You could use these pages to structure your introductory conversations.

Explaining the process

People will know less than you think about the investigation process. **Patients and family members** might have read the appropriate sections of their information booklet, but this is general information about what to expect. **Healthcare staff** might have experienced the process before, but even if they have we know each investigation is different. It is important that you set out the way you expect the investigation will happen. **Check how people would like to receive this information.**

- **Explain the general investigation process.** This might be a general discussion about the Trust investigation policy, or a more specific discussion about how you plan to complete the investigation.

- **Explain anything you have already done.** You might already have started the investigation. If you have, explain what you have already done and why it was important for you to do this before you involved different people in the process.

- **Ask if they have any questions about the investigation process.** If you can't answer certain questions, be honest and try and find out the answer or direct them to someone you think will be able to provide an answer.

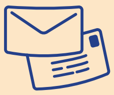

Could you send people a plain English summary and then follow up with them to check their understanding? Talking through the whole process might be a lot of information for someone to take in.

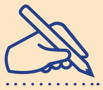

Could you write down key points in the investigation process to discuss verbally? This might help you to remain clear and concise if you have notes to guide you.

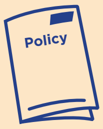

Do you have a copy of the Trust policy and the PSIRF available before the conversation? This might be the point at which patients, family members or staff members ask for a copy of these documents, or ask you questions about them.

Discussing support needs

Everyone will respond differently to incidents, and some people will be more aware of their support needs than others. Not everyone will need further support. It is likely, however, that support needs might change over the course of the investigation. It is important to understand these needs so that you can respond to them appropriately.

- **Do they need support?** People might not be aware of what they need, especially if the incident has been particularly traumatic. Try and prompt them with examples of support, using your expertise from previous investigations, or your understanding of the incident. Give them time and space to answer if they need it.
- **If so, what type of support?** Support needs can manifest in different ways. They can be *emotional*, *practical* or *psychological*. You should try and prompt people to think about these different types of support if they haven't already.
- **Sharing support resources.** If you have compiled support resources you think might be useful to them in your preparation for the investigation, you should offer to give them these, or discuss the organisations and what they offer so they can decide which would be most appropriate for them.

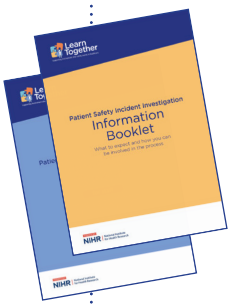

There is a **support section** on **pages 40-43** of the information booklets for people to note down details of the organisations you give them. You can prompt them to use this section if you are providing them with support.

There is also space for people to write down support they feel they need. You could ask if they have written anything in this section during your conversations.

Scheduling continued contact

You should discuss continued contact in these early communications. It might feel particularly daunting for patients, family members and healthcare staff to think about contacting you in an unstructured way. You should talk about whether people would prefer to have regular contact from you.

- **Ask if they would like regular contact with you during the investigation.** You could suggest weekly or bi-monthly contact for example, based on the investigation and the time you can dedicate to this. Be clear about how much time you have.

- **If they would like regular contact, discuss the best form of communication.** Some people might not have easy access to email or video software. Find out how they would prefer to be contacted, and make any boundaries clear for example if you are unable to contact them by text or other instant messaging.

- **Ask if there are any dates they don't want to be contacted.** There might be significant dates on which they don't want to talk about the incident or the investigation. If there has been a bereavement there might be a funeral date in the near future, around which they might find contact more difficult. Be clear about any holidays you have planned during the investigation phase, or any clinical commitments that might disrupt any regular communication.

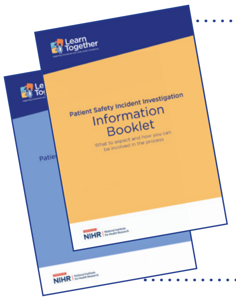

There is space on **pages 46-47** of the information booklets where people could record details of any contact, as well as details of any regular communication dates and times. You could suggest that people note agreed contact dates here.

Initial contact

Involvement preferences

Different people will want different levels of involvement in the investigation. Although these preferences might change as the investigation progresses, it is important to discuss them in your initial conversations. This will set clear expectations for you both about how and when they would like to be involved. You should discuss individual preferences for involvement in:

- **Terms of Reference**
- **Information gathering**
- **Draft report**
- **Final report**

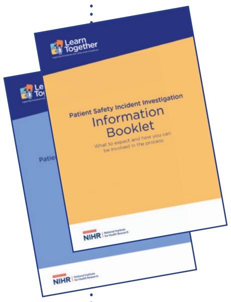

Have you read the information booklet descriptions of each point of involvement? It is important you know what people understand about each point of involvement so you can discuss each point and answer any questions they might have.

The information booklets also include a checklist on page 21 that can be completed to indicate when they want to be involved. You could use this checklist to structure your conversation.

Make it clear that preferences for involvement can change as the investigation progresses. Let people know that they can change their mind at any time, and how they should contact you to let you know if this happens.

Notes

Continued contact

Through your initial conversations you will have set the tone and established ways of working with different people involved in the investigation. As the investigation progresses, you will enact these different ways of working to enable those who want to, to engage in the investigation process. Continued contact will also mean being open and clear about how the investigation is progressing.

It is important as the investigation progresses to recognise that it is difficult for people to engage meaningfully with a system or process that they are unfamiliar with. **During your continued contact with people, you are providing both the opportunity and support for them to access the parts of the investigation process they want to be involved in.**

- **For people who want to be involved in the investigation,** your continued contact with them will be very similar to your initial conversations: you will provide information, discuss experiences and pay attention to support needs.
- **For people who don't want to be as involved, or involved at all,** continued contact might be as simple as giving simple updates or providing basic information. Different people might change their involvement preferences over the course of the investigation, so remaining flexible during your continued contact with people is essential.

As you enter into a new phase of communication it is important to attend to these principles for involvement:

Individualise your approach

Although you will have set expectations for continued contact with different people, involvement should be flexible and respond to potentially changing needs. People might change their mind over time about their involvement preferences.

Be sensitive to timing

You should be aware of any potentially sensitive dates following your initial communication. Time can feel distorted for people during times of stress or anxiety, so people might think the investigation is moving slowly or too quickly.

Listen

Everyone involved in the incident should have the opportunity to share their experiences of it. You can demonstrate a commitment to learning through active and engaged listening to these experiences.

Be collaborative and open

Openness and collaboration will make people feel involved in the investigation. When people feel they are being as involved as they want to be, they are more likely to feel positive about the process and the outcomes.

Accept subjectivity

People might want to talk to you about their experiences so that you can use this information within the investigation. It is important to accept that different people will experience the same incident in different ways, and that there is no one 'objective truth'.

On the following pages you will find ideas about how to make the most of your continued communication.

What to cover

Different people will have different communication requirements. You will have discussed their preferences for involvement, so you can be flexible with these ideas based on how involved they want to be.

- [ ] Maintain contact as initially agreed.
- [ ] Explain the proposed **Terms of Reference** and explore other questions.
- [ ] If requested, support them to share their experience of the incident.
- [ ] Ask if and how they want to receive the draft report.
- [ ] If requested, support them to discuss the draft report.
- [ ] Discuss any potential support needs.
- [ ] Revisit preferences.

Maintaining contact

Although you might not always have something to update people about, it is important that you are reliably maintaining contact. If for any reason you can't maintain your communication as agreed, you should let people know as soon as possible and arrange another mutually convenient time to contact them. It is also important to be transparent about how much progress you have made since the last time you spoke. By maintaining contact and being open, you will continue to build trust with the patients, family members and healthcare staff involved.

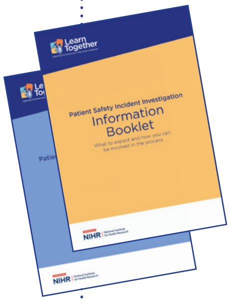

Have you checked if they have any questions? Patients, family members and staff might still lack confidence. You can continue to invite involvement by checking if they have any questions when you speak to them.

The information booklets include a **question log** on **pages 44-45**. You could refer to this as a way to check if people have any questions when you speak to them.

Terms of Reference

Setting the Terms of Reference is a key part of the investigation process. **Patients and family members** might have read the appropriate section of their information booklet that contains general information about what the Terms of Reference are and why they are important. **Healthcare staff** might have experienced the process before, but each investigation is different. It is important that you share the Terms of Reference with anyone who wants to see them.

- **Ask if and how people want to receive the Terms of Reference.**

- **Direct them to their information booklet, which includes information about what the Terms of Reference are.**

- **Explain how you decided on the Terms of Reference.** The information booklets make it clear that the Terms of Reference are a guide for you about what to focus on during the investigation. People might want to understand why you have decided on these terms.

- **Ask them if they have any questions that the Terms of Reference don't cover.** Listen to their questions. Be open about whether you think you will be able to answer their questions within the terms of the investigation or not.

- **Support people to access different sources of information.** If their questions lie outside of the scope of the investigation, support them to find people or organisations that could help them to answer these.

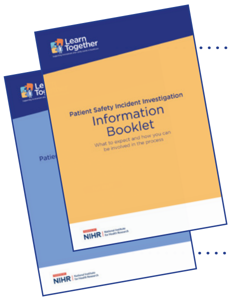

The information booklets have dedicated space on **pages 28-29** to record the Terms of Reference, and any additional questions. You could use this page to structure your discussion.

Continued contact

Sharing experiences of the incident

You might already have your own ideas about how and why the incident happened from any information you have received up to this point. Based on your experience as an investigator, any clinical understanding of the incident and your contextual knowledge about the Trust, this is **your subjective view** of what happened.

Patients and family members might not have the same level of clinical or contextual understanding as **healthcare staff.** However, everyone will have their own lived experience of the care leading up to the incident, what led to the incident and are often living with emotional, psychological or physical consequences.

It is important to consider all subjective experiences, and use them to arrive at a version of events that tries to represent these combined experiences.

Could you have this conversation on a video call or face-to-face to make it easier to respond to people as they share their experience?

Could you offer people the opportunity to have someone with them while you have this conversation? They might welcome the opportunity for support.

If people are sharing their experiences verbally, can you arrange this when you have more time or flexibility in your diary?
You should give people the time and space to share their experience with you in full. If you only have a specific amount of time, be honest about this and offer to arrange a second conversation.

- **Ask how they would prefer to share their experience.** If people feel comfortable, they are more likely to feel able to recall and share everything they remember about the incident and the events that surrounded it.

- **Explain how you will use the information they provide.** It is important to be transparent about how you will use this information. Sharing their experience might be difficult, emotional and daunting. Providing as much clarity as possible about how you will use this information will make them more comfortable.

- **Thank them for sharing their experience with you.** It is important to recognise how difficult an experience this might be, and how invaluable a contribution they have made to the investigation process.

- **Check whether they need any immediate support.** In the 24 to 48 hours following the patient, family member or member of staff providing you with an account of their experiences, you should check whether they need any additional support. You could use the support resources you gathered at the beginning of the investigation.

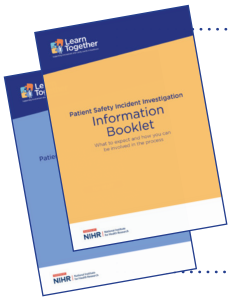

The information booklets have dedicated space on **pages 30-31** to write notes about key information they might want to share with you. You could suggest they use this page to structure their account of the incident.

Draft report

You will have a good idea of what the report will contain and of the recommendations you are going to make. Involvement at this stage might feel daunting, but having the chance to check the draft report for inaccuracies and to ask questions, will make patients, family members and healthcare staff feel that they are more equal partners in the investigation process. This is especially true when the draft report is checked internally, by members of the Trust governance, safety or legal teams or by the board.

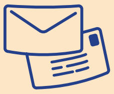

Could you send the draft report over email or by post, and then arrange time to discuss it? People will need time to read the draft. By arranging a verbal discussion, you can more easily set boundaries about what people can comment on, and clearly explain what you might and might not be able to change before the final report.

- **Ask how they would like to receive a copy of the draft report.** Be clear if you are unable to send the report in certain ways before it is finalised.
- **Be clear about what people are able to comment on.** This will set clear expectations. You could send a brief template that highlights particular areas to pay attention to or gives them some prompts to focus their comments.
- **Explain how you will use their comments.** If you can't change certain things, be honest and explain why not. People might not be aware of the organisational processes involved at this stage.
- **Explain what will happen between this and the final report.** Be clear about how you will develop the final report from this draft, and how similar people can expect the two to be.

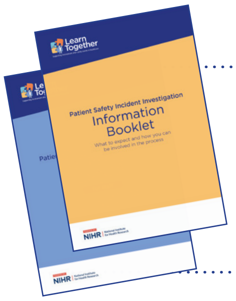

The information booklets have dedicated space on **pages 32-33** for people to note down any comments they have about the draft report. You could ask them to share these comments.

Discuss potential support needs

It is likely that support needs might change over the course of the investigation. Discussing potential support needs should be an ongoing conversation with people.

- **Do they need support?** People might not be aware of what they need, especially if the incident has been particularly traumatic. Try and prompt them with examples of support, using your expertise from previous investigations, or your understanding of the incident. Give them time and space to answer if they need it.

- **If so, what type of support?** Support needs can manifest in different ways. They can be *emotional*, *practical* or *psychological*. You should try and prompt people to think about these different types of support if they haven't already.

- **Sharing support resources.** If you have compiled support resources you think might be useful to them in your preparation for the investigation, you should offer to give them these, or discuss the organisations and what they offer so they can decide which would be most appropriate for them.

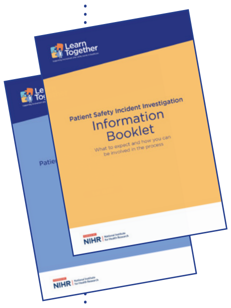

There is a **support section** on **pages 40-43** of the information booklets for people to note down details of the organisations you give them. You can prompt them to use this section if you are providing them with support.

There is also space for people to write down support they feel they need. You could ask if they have written anything in this section during your conversations.

Revisit involvement preferences

Different people will want different levels of involvement in the investigation. These preferences might change as the investigation progresses. You should check that people are still happy with the preferences they highlighted earlier in the investigation.

How might you check individual preferences? For people who did not want to be involved in the investigation, you should use your discretion about how to revisit their preferences. If you think they might have been unsure initially, you could send a brief email or a short letter to check that they are still happy. For those people who are involved in the investigation and in some form of continued communication with you, you can raise this during one of your regular communications.

Depending on the progress of the investigation, you should discuss individual preferences for involvement in:

- **Terms of Reference**
- **Information gathering**
- **Draft report**
- **Final report**

It is important that people can contribute to each of these stages of the investigation in some way until the final report is written, even if this is just a short discussion with you.

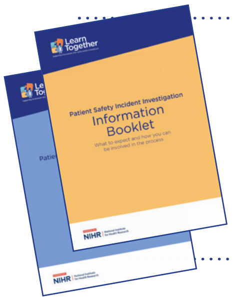

The information booklets include information about each of these key parts of the investigation, and a checklist of preferences for involvement on **page 21**. You could use this checklist to structure this conversation.

Make it clear when the final opportunity for contribution to each of these key parts of the investigation might be. This sets clear expectations and equal opportunity for everyone involved.

Notes

Closing contact

The end of the investigation is a point of closure for everyone involved. This might be an emotional process for people, regardless of their level of involvement during the investigation process.

The end of the investigation for you might mean you move on to another investigation or that you go back to full time clinical or operational work. Your role investigating this specific incident is complete.

For patients, families and healthcare staff, the end of this investigation might be the beginning of a new normal. **Patients and family members** might have to learn to live with the impact of the incident, whether that is physically, emotionally or psychologically. They might have to navigate additional healthcare services, or process life-changing injuries or bereavement. **Healthcare staff** might have to process feelings of guilt or might question their own professional ability. They might be nervous about what people think of them, or about another incident happening while they are on shift.

The investigation might have provided structure for people during a difficult time. It is important that closing contact is as positive an experience as it can be, and that you recognise the potential impact of the end of the investigation on everyone involved.

As you enter into this phase of communication it is important to attend to the following principles for involvement:

**Respect humanity**	Different people will have different emotional responses to the end of the investigation process. It is important to accommodate these different responses when planning how to close contact with different people.
**Treat people with compassion**	It is important to close contact in an empathic way to reduce the likelihood of compounding any harm caused by the incident.
**Strive for equity**	Organisational learning is the desired outcome for any investigation. But it is important to consider the impact of the end of the investigation on everyone involved.
**Individualise your approach**	Different people will need different levels of support at the end of the investigation. You should be flexible and adapt to these different needs.

On the following pages you will find ideas about how to sensitively close communication with patients, family members and healthcare staff at the end of the investigation process.

What to cover

You will have had different levels of communication with different people throughout the investigation. By this stage you will be more aware of their individual needs and preferences, and how they might feel as the investigation comes to a close. The following checklist should provide you with ideas about how you can close the investigation respectfully and sensitively.

- [ ] Maintain contact as initially agreed.
- [ ] Ask if and how they want to receive the final report.
- [ ] If requested, send them the final report .
- [ ] If requested, discuss the final report and the conclusions with them.
- [ ] If necessary, discuss any further investigations that might happen.
- [ ] Explore opportunities for further involvement where appropriate.
- [ ] Discuss the formal end of the process.
- [ ] Signpost them to any ongoing support services.

Maintaining contact

Although you might not always have something to update people about, it is important that you are reliably maintaining contact. If for any reason you can't maintain your communication as agreed, you should let people know as soon as possible and arrange another mutually convenient time to contact them. It is also important to be transparent about how much progress you have made since the last time you spoke. By maintaining contact and being open, you will continue to build trust with the patients, family members and healthcare staff involved.

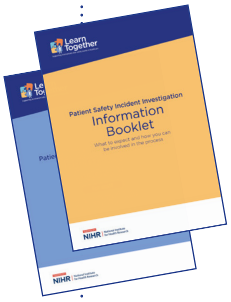

Have you checked if they have any questions? Patients, family members and staff might still lack confidence. You can continue to invite involvement by checking if they have any questions when you speak to them.

The information booklets include a **question log** on **pages 44-45**. You could refer to this as a way to check if people have any questions when you speak to them.

The final report

Receipt of the final report will mark the end of the investigation process for everyone involved. Make it clear when people can expect a copy of the final report so that receiving it isn't a surprise.

- **Ask how they would like to receive a copy of the final report.** It is important that the report is delivered in the easiest format for people to access.

- **Ask if people would like the opportunity to discuss the final report.** The report is likely to be difficult to understand for some people. You should be prepared to explain the meaning of the report, and answer any questions people might have.

- **Explain how the Trust will use the report.** Tell people the process of response to an investigation report. It is important that they can see how the Trust will respond to the incident.

Could you have this conversation on a video call or face-to-face to make it easier to respond to people when they provide comment on the final report?

Could you offer people the opportunity to have someone with them while you discuss the final report? They might welcome the opportunity for support.

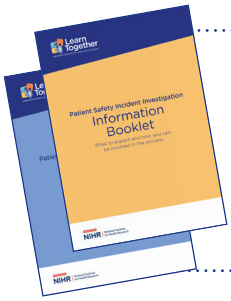

The information booklets have dedicated space on **pages 36-37** for people to summarise what is included in the final report, and any questions they have. You could ask them to share these comments to structure this conversation.

Further investigations

Although the final report will mark the end of the Trust-level investigation process, there might be additional investigation processes that are ongoing or that follow the investigation you have been leading. Although you might not be involved in any further investigations, it is important that you prepare people if you know of any processes that will occur from this point, such as an inquest.

Could you incorporate this topic into your discussion about the final report? When you explain how the Trust will respond to the recommendations in the final report, you could explain that another response to the end of this investigation is the beginning of another investigative process.

Depending on your previous experience, and on how much information you have, you might only be able to give basic details. **Provide people with as much information as you can, but be honest if you don't know very much about the additional investigation processes.** If you know colleagues who might have more information, you could direct people to them with their permission and if you feel this is appropriate.

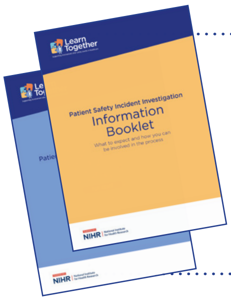

Page 38 of information booklets includes information about the coroner, inquests, and organisations like the **Healthcare Safety Investigation Branch (HSIB)**. You could direct people here for more information.

Opportunities for further involvement

Some patients, family members or members of staff might provide valuable information about how they think the Trust might improve certain processes to reduce the likelihood of incidents happening again. It will be important to use your professional discretion at this stage, but if you think certain people might be able to support the Trust with specific ideas for change or improvement, you could offer them opportunity for further involvement.

- **Who would you talk to at the Trust?** This won't be a unilateral decision for you to make. You should be clear about who you could talk to at the Trust if you think someone might be interested in the opportunity for further involvement in the response to the report. You should develop a clear plan about who would support their involvement, and who would be their main point of contact going forward.

- **Would people still have access to support?** Although people who agree to support the Trust are likely to feel positive about the opportunity for further involvement, you should make sure there is a plan for continued access to support.

- **How would their relationship with the Trust formally close?** Although people might be positive about the opportunity for further involvement, you should make sure that any Trust colleagues they work with set out a clear plan for their involvement. A lack of formal closure might lead to people feeling used or feelings of additional harm following the incident.

Formal end of the process

It is important to formally close the investigation. Different people will have had different levels of involvement in the investigation, so your formal closure might differ depending on this.

For people who *have* been involved during the investigation, you will be able to close communication during a regular point of contact. You should thank them for their involvement in the investigation.

For people who *haven't* been involved in the investigation, it is important that they are still made aware that the investigation has officially finished. You might want to send them an email or a letter.

When closing contact with anyone, regardless of their level of involvement in the investigation, you should recognise the impact of the incident and the investigation.

Give another meaningful apology for the incident, and reassure them that the Trust is committed to responding to the outcome of the investigation to try and reduce the likelihood of similar incidents happening in future.

You might want to outline how the Trust might respond to the outcome of the investigation if you know.

Ongoing support

It is likely that support needs might change over the course of the investigation. Whilst some people will not have support needs, for others the end of the investigation might be a particularly emotional time.

- **Do they need support?** People might not be aware of what they need, especially if the investigation has been difficult or emotional. Try and prompt them with examples of support, using your expertise from previous investigations, or your understanding of the incident. Give them time and space to answer if they need it.

- **If so, what type of support?** Support needs can manifest in different ways. They can be *emotional*, *practical* or *psychological*. You should try and prompt people to think about these different types of support if they haven't already.

- **Sharing support resources.** If you have compiled support resources you think might be useful to them in your preparation for the investigation, you should offer to give them these, or discuss the organisations and what they offer so they can decide which would be most appropriate for them.

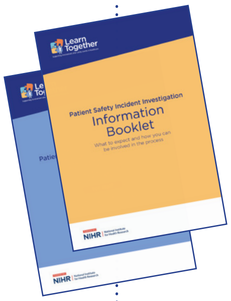

There is a **support section** on **pages 40-43** of the information booklets for people to note down details of the organisations you give them. You can prompt them to use this section if you are providing them with support.

There is also space for people to write down support they feel they need. You could ask if they have written anything in this section during your conversations.

Support for you

You might value support to process details of the incident, what you have heard during the investigation, or support after taking on the emotional load of those involved in the investigation. Below are the details of some different organisations you can access for support and guidance.

You might find that you would benefit from more specific support related to the incident, or more practical support with the investigation process. We encourage you to speak to your line manager, or trusted colleague in the first instance, if you require more specific or locally appropriate support. You can also speak to the Occupational Health team in your Trust confidentially or access support through membership of any professional organisation.

NHS staff support line is a confidential, free-phone line operated by the Samaritans. It is available to access between 7am and 11pm, seven days a week. You can call the dedicated staff support line if you would like to talk to someone confidentially if you have had a hard day, if you are worried about anything, or if you just feel you would like to talk to someone. The free-phone line number is **0800 069 6222**. Alternatively you can text **FRONTLINE** to **85258** for text support. Text support is available 24 hours a day, seven days a week.

The NHS staff wellbeing hubs have been set up to provide NHS staff access to local support and mental health services where needed. The hubs can help you access support such as talking therapy and counselling. You can self-refer to your local hub. There is more information about the Hubs and how to access them at **https://www.england.nhs.uk/supporting-our-nhs-people/support-now/staff-mental-health-and-wellbeing-hubs/**

Your Local Clinical Commissioning Group (CCG) are responsible for monitoring the serious incident investigations and reports from your organisation. The commissioners might be able to provide you with support if you have queries or concerns about the outcome of the investigation, or how the actions and recommendations might be applied in your Trust. You can find out about your local CCG and their contact details at **www.england.nhs.uk/ccgs/**.

Key words and phrases

Case note review: Use of case or medical notes to determine whether there were any problems with the care provided to a patient, identify the prevalence of issues, or when families/carers or staff raise concerns about care.

Clinical audit: Measurement of the effectiveness of specific elements of healthcare against agreed and proven standards for high quality, with the aim of then acting to bring practice into line with these standards to improve the quality of care and health outcomes.

Clinical Commissioning Group (CCG): CCGs are groups of general practices (GPs) who come together in each area to commission the best services for their patients and the local population. This means that they continuously plan, agree on and monitor NHS services in the local area. **https://www.england.nhs.uk/commissioning/**

Commissioner: Commissioners might include people who have been GPs, or other clinicians such as nurses and consultants. Commissioners will sit on governing bodies which might also include patient representatives, general managers and practice managers.

Common themes: Common themes are recurring ideas, subjects or topics, relevant to the incident and the **Terms of Reference**, the **investigator** identifies when they are reading all of the information they have collected about the incident.

Her Majesty's Coroner: A coroner is a government official or member of the judicial system who carries out **inquests**.

Department for Health and Social Care (DHSC): The government group responsible for health and social care across the UK.

Duty of Candour: A professional responsibility for healthcare staff and organisations to be honest with patients and families when things go wrong. The patient and/or family should be told when something has gone wrong, should be offered an apology and appropriate support, and the full effects of what has happened (if any) should be explained.

Family liaison officer (FLO): A member of Trust staff whose primary role is to provide compassionate support and advice to patients and their families during a **patient safety incident investigation**.

Governance team: Governance teams work in NHS organisations and are responsible for monitoring the quality of services and for safeguarding high standards of care.

Healthcare incident: Any unplanned or unintended event or circumstance which could have resulted or did result in harm to a patient.

The Healthcare Safety Investigation Branch (HSIB): HSIB is funded by the **Department for Health and Social Care**, and is responsible for carrying out independent investigations into NHS-funded care across England.

Hot debrief: A post-incident review by the medical team used to collectively discuss and answer a series of questions.

Incident response lead: A member of staff, normally employed by the healthcare provider, who has been trained to conduct **patient safety incident investigations**. For example, this might be the Trust **patient safety specialist**.

Inquest: An inquest is a formal investigation conducted by a **coroner** to determine how someone died.

Learning disabilities mortality review (LeDeR): A specialist review of the care of a person with a learning disability (recommended alongside a **case note review**).

Legal team: Most NHS Trusts have legal teams to manage a wide range of legal matters for the Trust including: claims brought against the Trust; **inquests**; any proceedings involving Trust witnesses; medical treatment applications to the High Court; medical records requests from solicitors.

Mortality review: A review of a series of case records to identify any problems in care and draw learning or conclusions that inform action needed to improve care, within a setting or for a specific patient group, particularly in relation to deceased patients.

Near miss: An event that does not cause harm, but which has the potential to cause injury or ill health if it had not been caught in time.

Never event: Serious, largely preventable patient safety incidents that should not occur if the available preventative measures have been implemented.

Patient safety incident: Any unintended or unexpected incident which could have, or did, lead to harm for one or more patients receiving healthcare.

Patient safety incident investigation: These are investigations conducted to identify how and why certain patient safety incidents happen. They are not inquiries into the cause of death or for apportioning blame. Investigations result in a set of recommendations and an improvement plan that is designed to effectively and sustainably address the underlying factors in the organisation that led to a patient safety incident, to help deliver safer care in the future.

Patient safety specialist: Individuals in healthcare organisations who have been designated to provide senior patient safety leadership.

Patient safety team: Most NHS Trusts will have a patient safety team dedicated to working within the service to minimise the risk and impact of incidents.

Perinatal mortality review: A specialist multidisciplinary audit and review to determine the circumstances and care leading up to and surrounding a stillbirth or neonatal death, and the deaths of babies in the postneonatal period having received neonatal care.

Policy: An official document that includes a set of guidelines to guide decisions and achieve specific outcomes.

Policy makers: In this context, we mean members of staff from the **Patient safety team** at NHS England and Improvement.

Public inquiry: Public inquiries are independent, national level investigations ordered by a government department to deal with matters of public concern.

Terms of Reference: These are guidelines that define the scope and purpose of the investigation.

YQSR Yorkshire Quality and Safety Research Group

NIHR | Yorkshire and Humber Patient Safety Translational Research Centre

NHS Bradford Teaching Hospitals NHS Foundation Trust

UNIVERSITY OF LEEDS

This material was developed as part of the Patient and Family Involvement in Serious Incident Investigations (PFI SII) research study. The materials were co-designed by the PFI SII research team, Lab4Living and a community of patients, families, healthcare staff and managers.

Lab 4 Living

FUNDED BY

NIHR | National Institute for Health Research

This study is funded by the National Institute for Health Research NIHR (Health Services and Delivery Research; NIHR Reference Number 18/10/02). The views expressed are those of the authors and not necessarily those of the NIHR or the Department of Health and Social Care.
