# Supplementary material for: The Learn Together programme (part A): co-designing an approach to support patient and family involvement and engagement in patient safety incident investigations
Source: Front Health Serv. 2025 Mar 26;5:1529035. doi: 10.3389/frhs.2025.1529035 (PMC11979208; doi:10.3389/frhs.2025.1529035)
Supplement: Supplementary file 3 [file Datasheet3.pdf]

Learn Together

Supporting involvement after safety events in healthcare

Patient Safety Incident Investigation

Information Booklet

What to expect and how you can be involved in the process

FUNDED BY

NIHR | National Institute for Health Research

My main point of contact

Name: ....

Contact info: ....

....

© Copyright Bradford Teaching Hospitals NHS Foundation Trust, 2021. All rights reserved. This material must not be reproduced or shared without permission from Professor Jane O’Hara (jane.o’hara@bthft.nhs.uk).

Introduction

Why am I receiving this booklet?

You have recently experienced a healthcare incident that will be reviewed by the NHS Trust where it happened as part of a **patient safety incident investigation**. We know this might feel daunting. This information booklet has been designed in the hope that it will help make the investigation process easier and more meaningful for you.

A patient safety incident investigation is something an NHS Trust does to learn about what happened, why it happened and how things can be changed or improved to try and make sure it doesn't happen again. We know you probably want to know these things too. This booklet will give you the information you need to be involved throughout the investigation process if you would like to be.

This is your booklet to keep. You won't have to return it after the investigation, and you do not need to share anything you write in it unless you want to.

What do I need to know now?

The investigation is a bit like a jigsaw. There are different people who might have experienced the incident. A good investigation tries to understand those different people's experiences of the incident.

You are a key part of this jigsaw. The investigation is an opportunity for you to shape what the incident response lead looks into, and to learn more about what happened and why.

We know that the investigation can't change what happened, but we hope it will help you to learn about what happened and the ways in which the Trust are committed to change and improve from this point.

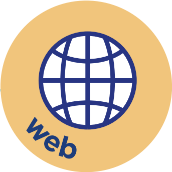

The key information you might need during the investigation is in this booklet. We will also highlight websites or pages that will supplement the information we provide, or that you can access for more general support.

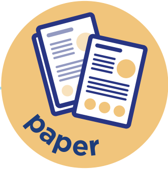

If you cannot access the internet, or if you prefer to have everything on paper, we have highlighted where paper copies of documents are available throughout the booklet. Wherever you see the symbol above, you can ask the incident response lead for a paper copy of the document highlighted.

Contents

This information has been designed to help guide you through the patient safety incident investigation process. The design process involved patients, family members, healthcare staff, investigators and policy makers who have all been involved in incident investigations in healthcare.

The booklet is in two parts. A detailed contents page can be found at the beginning of each section.

General information 7

This section contains all of the key information you might need about patient safety incident response and the patient safety incident investigation process. This should give you a good idea of what you might expect during the incident investigation you are involved in.

Your investigation 19

This section is specific to your investigation. It has been designed to support you to be as involved as you would like to be. It includes space for you to record key information, any questions you might have, and anything else you feel is important.

Key words and phrases 53

You'll notice that we've highlighted certain words in **pink** in this booklet. These are words or phrases that might be unfamiliar to you. We've collected the definitions in a list at the back of this booklet.

General information

The following section of the booklet has been designed to provide you with general information about what patient safety incidents and patient safety incident investigations are. We hope that this will prepare you for the investigation process, and help you understand what to expect as it progresses.

Patient safety incidents: A definition **8**

Patient safety incident investigations **10**

Identifying the response type **12**

Gathering information **14**

Writing the report **16**

Patient safety incidents: A definition

The NHS has its own definition of a patient safety incident. You've been given this booklet because the NHS Trust where the incident happened has decided that it fits this definition of a patient safety incident.

"Unintended or unexpected incidents which could have or did lead to harm for one or more patients receiving healthcare."

- Patient Safety Incident Response Framework (PSIRF)

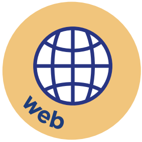

If you would like to read the PSIRF, you can find it here: **https://www.england.nhs.uk/patient-safety/incident-response-framework/**

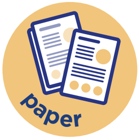

The PSIRF was written to guide all NHS Trusts in England in how to identify, and respond to patient safety incidents. Individual Trusts will have written their own **policy** based on this national document. **You can ask your incident response lead for a copy of the Trust policy if you would like to read it.**

Different Trusts might use different words or phrases to describe a patient safety incident, for example:

- **Adverse event**
- **Patient safety event**
- **Serious untoward incident**
- **Near miss**
- **Never event**

Patient safety incident investigations

NHS Trusts want to learn about why patient safety incidents happen, and how they can reduce the likelihood of the same incident happening in the future. The Trust will carry out a patient safety incident investigation to try and find out what happened and why.

The aim of a patient safety incident investigation is to understand the circumstances that led to the incident, and identify procedures, practices or areas in the system or organisation that might need to be changed or improved. It is not to determine cause of death or to blame an individual or hold an organisation to account. There are other channels for these processes where necessary.

You can find more detailed information here:

web

The **Patient Safety Incident Response Framework** - **https://www.england.nhs.uk/patient-safety/incident-response-framework/**

paper

The patient safety incident response policy of the NHS Trust where your investigation is happening.

What does this mean?

The incident response lead wants to find out what happened, and importantly why. They'll collect information to help them understand what happened. They will also consider what led to the incident, and the actions that can be taken to help prevent or significantly reduce the likelihood of a similar incident in the future. The incident response lead will write a report which explains what they found out. The report will always include the lessons learned and actions for the Trust to support them to achieve the intended change and improvement.

What does this mean for me?

You might have your own questions about what happened. The incident response lead should be able to answer some of these during the investigation. If any of your questions are outside the scope of what the incident response lead can find out during the investigation, they will explain why and try to find the right people to answer these.

The introduction of the Patient Safety Incident Response Framework (PSIRF) gives Trusts more ways of responding to patient safety incidents. You have received this booklet because you are involved in a patient safety incident investigation, which is the highest level of scrutiny a Trust can give to a patient safety incident. There are a number of other response types available to Trusts. The Trust might use some of these in addition to the main incident investigation, such as:

- **Case note review**
- **Hot debrief**
- **Learning disabilities mortality review (LeDeR)**
- **Perinatal mortality review**
- **Mortality review**
- **Clinical audit**

What does a patient safety incident investigation include?
There are three key parts to every investigation. Depending on the incident and the Trust investigation policy, these might happen one after another or they might overlap. The three key parts are:

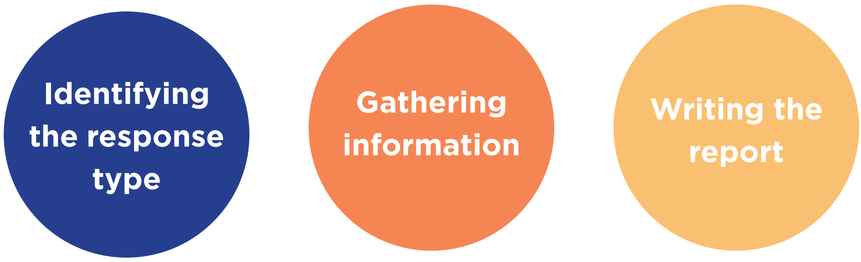

The following pages will break down these three key parts of an investigation. For each part we describe what might happen and why this is important to the investigation.

As the investigation progresses, you might want to make a note of what is happening or how you are feeling. You will find a timeline template on **page 48**. This is your space to record the investigation process, your involvement, your experiences and anything else you might find useful to keep a record of.

Rather than using a set template, you might find it easier to draw up your own timeline. We have also included a blank timeline template on **page 50**.

Identifying the response type

Response

The Trust considers the incident against national and local investigation priority areas to decide on the appropriate response type.

Duty of Candour

The Department of Health and Social Care mandates that the Trust makes a patient or their family aware of an incident as soon as they realise.

The NHS Trust will have identified this as a patient safety incident and will have used national and local documentation to decide that a patient safety incident investigation is the most appropriate response. This generally happens very soon after the incident itself, but depending on the circumstances it could be weeks or months afterwards. As soon as is safe and possible after the incident is identified, the Trust must let the patient or family know that an incident has happened. This is called **Duty of Candour.**

Trusts are legally required as part of **Department of Health and Social Care (DHSC)** policy to let patients and families know that an incident has happened through Duty of Candour. This means that the consultant in charge of care when an incident happens (or another member of staff where appropriate) must tell the patient or family member what the incident was as soon as possible.

You can ask the incident response lead for a copy of the Trust Duty of Candour policy if you would like to read it.

You can also find more information about the Duty of Candour process on the **Care Quality Commission (CQC)** website: **https://www.cqc.org.uk/guidance-providers/regulations-enforcement/regulation-20-duty-candour**

After the Trust have decided that a patient safety incident investigation is appropriate, the incident response lead will be appointed. The incident response lead will be a trained member of staff at the Trust. They will be independent of the clinical area in which the incident happened where possible and appropriate. At this point, the **scale** and **scope** of the investigation will be decided.

The **scale** of the investigation usually depends on how complex the incident has been and whether yours is the only incident being reviewed as part of this patient safety incident investigation. More complex incidents might involve more than one of the same type of incident, or a bigger team of investigators, for example.

The **scope** of the investigation is when the **Terms of Reference** are set. These act as a guide for the incident response lead and everyone else involved in the investigation by laying out what will be included, and the questions that need to be answered. The Terms of Reference also help the incident response lead to write the final report.

Gathering information

Reading relevant documents

Observation of clinical areas or procedures

Talking to patients, family and staff who were involved in the incident

Once the Terms of Reference are set, the incident response lead will identify the type of information they need and how they will collect it. The information gathered during this stage is what the incident response lead will use to explain what happened and why.

There are lots of different types of information an incident response lead will be able to use to answer the questions set out in the Terms of Reference. They might **review medical notes** to see what was written down about the incident at the time. Investigators might also **visit the ward or unit** where the incident happened, and **look at Trust policies or protocols** if they need to.

Information gathering might also involve **talking to the people who experienced the incident**. By talking to the different people involved, the incident response lead can build up a good idea of what happened and the impact of the incident.

Writing the report

Analysing Information

The investigator will search for **common themes** related to what happened and why.

Draft Report

The investigator sets out what they did and what they found out. They also draft an overview of the learning from the investigation, and actions for the Trust to prompt change or improvement.

Report Checking

The report might be sent to teams in the Trust like **governance** or **patient safety teams** as well as the Trust legal team.

The incident response lead will look through all of the information they have collected. They will use the information they have to piece together the most accurate version of the incident they can, taking into account different perspectives. By building up a good picture of what happened, the incident response lead will identify the different things that might have contributed to the incident. Incidents are very rarely caused by a single thing. Usually there are multiple reasons.

Understanding why the incident happened means the incident response lead can help the Trust learn about how to prevent this type of incident or reduce its likelihood. These actions might be developed with staff at the Trust to make sure they are achievable. There might be certain things that the Trust might not be able to do immediately (e.g. increase the number of staff, or change a particular working environment) but Trusts must attend to all actions outlined in a report and this will be monitored by local commissioners.

Once the incident response lead has written the **first draft of the report**, they might send this to different people to check. For example, they might want to make sure they have captured everyone's account of the incident properly. The incident response lead will then write up the **final report**.

Your investigation

This next section has been designed to support you to be involved during your investigation. It is divided into three sections; **initial conversations**, **continued contact** and **closing the investigation**.

At the end of this section you'll find spaces to record key information about the investigation so that you can keep it all in one place. There is also information to support the different ways you might be able to be involved if you would like to be.

The incident response lead, or your main point of contact if different, might refer to this section of the booklet in their initial conversations with you, and in ongoing communications if you decide to be involved in the investigation.

Initial conversations **21**

Key investigation staff details **22**

Incident details **24**

Continued contact **27**

Terms of Reference **28**

My experience **30**

Draft investigation report **32**

Closing of the Investigation **35**

Final investigation report **36**

Further involvement **38**

Your support needs **40**

Question Log **44**

People/ Organisations I've met **46**

Timeline templates **48**

Notes **52**

Initial conversations

The incident response lead (and the staff member who will be your main point of contact if different) will always aim to make contact with you as soon as possible once the investigation begins. Where possible this will be at the start of the investigation, but sometimes it might have already started when they make contact. In this first conversation they will introduce themselves to you. They might also talk to you about the incident and the investigation process. They will ask you if you want to be involved in the investigation, and/or have continued contact as it progresses.

Don't worry if you don't cover everything you would like to during your first conversation. The staff member(s) will arrange a follow up conversation.

Here's a list of ways you can be involved. Use this checklist in your initial conversations. You can change your mind about these at any time.

I would like...

- [ ] **Further contact about the investigation.**
- [ ] **To share my experience of the incident.**
- [ ] **To ask questions that I would like looked at in the investigation.**
- [ ] **To see a copy of the draft report.**
- [ ] **To see a copy of the final report.**
- [ ] **The opportunity to discuss the final report.**
- [ ] **Access to specific support organisations**

Key investigation staff details

It is important you know your main point(s) of contact during the investigation. You might find it helpful to record the staff member's details here when you first speak to them. You could also use this page as a prompt if you want to ask them any specific questions about their role or what their working hours will be if that would be helpful for you.

The incident response lead will be the person responsible for leading the patient safety incident investigation. They might be your main point of contact during an investigation, but you might have another nominated point of contact, such as:

- **Family Liaison Officer (FLO)**
- **Patient Safety Specialist**

You can get in touch with your main point of contact at any time during their working hours and if they are unavailable they'll get back to you as soon as they can. If you have any questions outside of working hours, you can note them down in the **Question Log** on **page 44**, or you can send them in an email and they'll get back to you as soon as possible.

As well as the person responsible for leading the investigation you are involved in, there might be other investigators working as part of a wider team. You might also meet other people during the course of the investigation. There is a place to log their details in the **People I Meet** section on **page 46**.

My main staff contact is:

Information about them and their role in my investigation:

....
....
....
....
....
....
....
....
....

Ways to contact the them:

Working hours:

....
....
....
....
....
....
....

My regular contact slot with my main staff contact is:

Incident details

This page is a space for you to note any important details about the incident if you would find this helpful. You might use this in your initial conversations with the incident response lead to record specific details about the incident that you would like to share with them.

There is no mandated time limit for a patient safety incident investigation, but they should average around 3 months and last no longer than 6 months. You should be involved in the conversations about how long the investigation will take.

The agreed endpoint for my investigation is:

Sometimes things might not happen as planned, but if your investigation is not going to finish within this timeframe, the incident response lead will tell you and will discuss a new timeframe with you.

The updated endpoint for my investigation is:

The patient safety incident that I was involved in was:

....

....

....

....

....

....

....

....

....

....

It happened on (DD/MM/YY):

Additional Notes (e.g. name of the hospital or care setting, ward or unit number, any additional details about the incident)

....

....

....

....

....

....

....

....

....

....

....

Continued contact

Your main point of contact for the investigation will have talked you through the checklist on **page 21** to understand if and how you would like to be involved during the investigation. For some, this might be a regular day and time to speak to your main staff contact for an update. Others, might also have expressed interest in being directly involved in specific parts of the investigation.

This next section will support your ongoing involvement. Here, you will find space to capture discussions with key staff including the incident response lead and important information on the key parts of the investigation you wish to be involved in. You might also find it useful to go back to information from part one of this booklet.

Use the list below to direct you to the sections you need:

To ask questions that I would like looked at in the investigation
Terms of Reference **28**

To share my experience of the incident
My experience **30**

To see a copy of the draft report
Draft investigation report **32**

To see a copy of the final report
Final investigation report **36**

The opportunity to discuss the final report
Final investigation report **36**

Access to specific support organisations
Your support needs **40**

Your main point of contact will record your preferences and support you to be involved in the investigation in the ways you would like to be. Your preferences for involvement might change during the course of the investigation. You can talk to your contact about being more, or less, involved at any time.

Terms of Reference

As outlined on **page 13**, the incident response lead will set the Terms of Reference to guide them in what to look at, and the questions that need to be answered during the investigation. The incident response lead will use their knowledge of the Trust, and their clinical expertise, if appropriate, to set the Terms of Reference.

Although the initial Terms of Reference for the investigation will be set by the incident response lead, or the investigation team, you will also be able to input if you would like to. The Terms of Reference will always be shared with you.

The Terms of Reference for my investigation are:

....

....

....

....

....

....

....

....

....

Any additional notes or thoughts about Terms of Reference:

....

....

....

....

....

....

....

Hopefully the Terms of Reference will cover most, if not all, of the questions you would like the investigation to answer. If you have any additional questions that are not included in the Terms of Reference, the incident response lead (or main staff contact) will direct you to different people or organisations that might be able to help you. You can include these details in the log on **page 46**.

The additional questions I have are:

....

....

....

....

....

....

....

....

....

....

....

....

....

....

....

....

....

....

....

....

....

My experience

As outlined on **page 14**, the incident response lead might speak to people who were involved in the incident about their experiences, to find out more about what happened and why.

If you would like to share your experiences with the incident response lead, you can use this space to note down the important points you would like to share with them.

Draft investigation report

You will be given the opportunity to see the draft report, or sections of it, if you would like to. For example, the incident response lead might share sections of the report where they outline your experience, or the impact the incident has had on you. The incident response lead may not be able to share the full report, but if this is the case they will explain why.

If you see the draft report, or parts of it, you can note down any inaccuracies you notice in the space below, or any sections you would like to ask the incident response lead about:

Closing the investigation

As the formal investigation process comes to an end, you might find that this is a difficult and emotional time. During this stage you will receive the final investigation report. This tells you what the incident response lead concluded about how and why the incident happened. You might also like to talk directly about the end of the investigation and how you feel about this. The incident response lead will ask you about any support you might need moving forward, and they will help you to organise this if you would like them to.

The following pages include space to note the key details about the final investigation report, and will give you some ideas about different ways of managing the end of the investigation. There is also space for you to note down details of any support you might need, or contact details of support organisations that your incident response lead or main point of contact directs you to. We have provided contact details of more general support organisations, and information about what to do if you aren't happy about the conclusion of the investigation.

Final investigation report

You will receive a copy of the final investigation report. Because the purpose of the investigation is to help the Trust learn about what happened and why, some of the specific language or phrasing used in the report might be unfamiliar.

The incident response lead will give you the chance to go through the report with them. If you would like to do so, they will talk you through what they found and how they decided on their suggested actions for the Trust.

You can record the important details from the investigation report here in your own words.

I received a copy of the investigation report on (DD/MM/YY):

The important parts of the final report were:

....

....

....

....

....

....

....

....

....

....

....

....

....

....

....

Further involvement

The end of the formal investigation process might feel difficult, whether you have been involved in the investigation or not. Your main point of contact from the Trust will talk to you about any further investigation processes that might occur following this one, and different opportunities for further involvement if appropriate.

Other investigations

In most cases, the end of this investigation process will mean that conclusions have been made about what happened. The Trust will be responsible for responding to the action plan set out in the final report. However, there are cases in which further investigation processes are necessary. These might happen at the same time as the Trust investigation, or they might happen afterwards. For example, following an unexplained death there will be an **inquest** led by the **coroner**, and following certain incidents in maternity services the **Healthcare Safety Investigation Branch (HSIB)** might carry out an independent patient safety incident investigation.

If the incident response lead knows there is going to be another investigation alongside or immediately after this one, they will tell you. They will also support you to find out more information about the additional investigation process if you would like to know more.

There will be an additional investigation of this incident by:

You can record any notes about the additional investigation process here:

....

....

....

....

....

....

....

Opportunities for further involvement

Once the final investigation report is complete, this marks the end of the formal investigation process. For the Trust, this marks the point at which they must start to implement the action plan included in the report.

It is important that they have support and guidance to do this. An organisation called the **Clinical Commissioning Group (CCG)** employ commissioners who monitor the actions from patient safety incident investigation reports. Trusts are expected to address these actions within specific timescales, set by the commissioners based on how complex they are.

Web

You can find more information about the CCG, and links to the local CCG in your area, on the NHS website: **https://www.england.nhs.uk/ccgs/**

The Trust will have specific processes that they use to respond to actions and these might be planned with the commissioner. However, there may be instances where you are offered the opportunity to meet with the members of staff at the Trust responsible for responding to the action plan. This might be more likely if you have specific information about ways the service could be changed or improved based on your experience of the Trust, and of the incident itself. **This will not always be possible, but you can speak to the incident response lead if this is something you would be interested in.**

If there are no opportunities for further involvement in the Trust, you might find it useful or cathartic to become involved more generally in supporting others who have experienced an incident or who are going through the investigation process. There are a number of advocacy groups, support groups and charities who would welcome your experience. **If this is something you are interested in, you should speak to your incident response lead (or main point of contact). They might not know immediately of any groups or organisations, but they will support you to find out more.**

Your support needs

Here are some general support resources that might help you if you have been affected emotionally by the incident you were involved in, or if you are finding the investigation process difficult.

Citizens Advice can give high quality, independent advice about any problems or questions you might have. They can provide you with the knowledge and confidence to find a way forward. They have a network of national and local independent charities that can provide free and confidential advice. You can find out more at **https://www.citizensadvice.org.uk/**. You can also call an advisor on **0800 144 8848.**

Mind is a registered charity who provide support and advice to anyone who is suffering with their mental health. If you have been affected emotionally following the incident you experienced or if you are finding the investigation process difficult, you can contact Mind. You can find general support resources and information about local services at **https://www.mind.org.uk/**. You can also email them on **info@mind.org.uk** or call their helpline on **0300 123 3393.**

Samaritans are a registered charity providing support to anyone in emotional distress or anyone who is struggling to cope. If you have been emotionally affected by the serious incident you were involved in, you can contact the Samaritans for free and there will always be someone there to listen to you and talk to you. You can find more information at **https://www.samaritans.org/**. You can email them on **jo@samaritans.org** or you can call free on **116 123**. Their support is available 24 hours a day, 7 days a week, 365 days of the year.

You might benefit from specific support related to the incident, or how it has affected you. You can discuss this with your main point of contact during the investigation. They will have a list of specific support organisations and can provide you with the details if you need them.

Details of specific organisations that can help me:

....

....

....

....

....

....

....

....

....

Additional Support. You can use this space to note down any support you need below. You can add to this during the investigation, and you can discuss this with your incident response lead or main point of contact..

....

....

....

....

....

....

....

....

....

....

What if I'm not happy?

The investigation report should have answered most, if not all, of the questions you had about how and why the incident happened. However, in some cases you might still have questions that the incident response lead was not able to include during the investigation, or questions that have arisen after reading the final report. These are details of different organisations you can access for support and guidance.

Action against Medical Accidents (AvMA) is a registered charity which aims to support people affected by avoidable healthcare harm. They can provide specialist, independent advice and support following serious incidents in healthcare. **www.avma.org.uk**

Patient Advice and Liaison Service (PALS) can be found in most hospitals. You can talk to a PALS member about your incident and they will try to help you resolve any issues with the Trust informally. PALS can be particularly useful if you need action immediately, for example if the incident has been a problem with your care and you are still in hospital. You can ask a member of staff at the Trust for details of the local PALS service, or you can find more information at:

www.nhs.uk/nhs-services/hospitals/what-is-pals-patient-advice-and-liaison-service/

If you want to make a formal complaint, you can go through the **NHS Complaints Service**. You can either make a complaint to the NHS Trust directly, or you can make a complaint to the local **Clinical Commissioning Group (CCG)** who oversee hospital services. You can't make complaints to both.

If you make a complaint to the NHS Trust directly, you can find details about who to contact on the Trust website or from a member of Trust staff.

If you make a complaint to the CCG, you can find out about your local CCG complaints procedure at **www.england.nhs.uk/ccgs/**. There is more information about making a complaint at **www.nhs.uk/using-the-nhs/about-the-nhs/how-to-complain-to-the-nhs/**. You can ask for support with the complaints process from your local NHS Complaints Advocacy Service. Your local council will be able to tell you who your local advocacy service is and provide their details.

The Parliamentary and Health Service Ombudsman (PHSO) respond to unresolved complaints. They can support you if you have made a complaint following your incident and the organisation has not responded to your complaint or you are dissatisfied with their response. You can find more information at **www.ombudsman.org.uk.**

Question log

Date	My question	Response

Question log

Date	My question	Response

People I have met

During the investigation process you might only meet and communicate with the incident response lead. Or there might be other people the incident response lead introduces you to, or that you meet as the investigation progresses. This is a space for you to capture the names of anyone else you meet during the investigation, what their role is, and how they are involved.

Name	Date met	Job Role	How they are involved

Organisations I have met

During the investigation process you might only have contact with the NHS Trust where the incident happened. Or there might be other organisations involved, perhaps because they were also involved in your care, or because they can help with the investigation. You can note details of any of these organisations below if you would like to keep a record of their involvement.

Name of organisation	Date they became involved	How they are involved

If you need more space to capture any of this information you can ask the incident response lead for extra pages.

Timeline template

As your investigation progresses, you might want to make a note of what is happening or how you are feeling at different points. You can log anything you find useful along the timeline. This is your own space to keep a record of the investigation progress, your involvement, your experiences and anything else you might want to record.

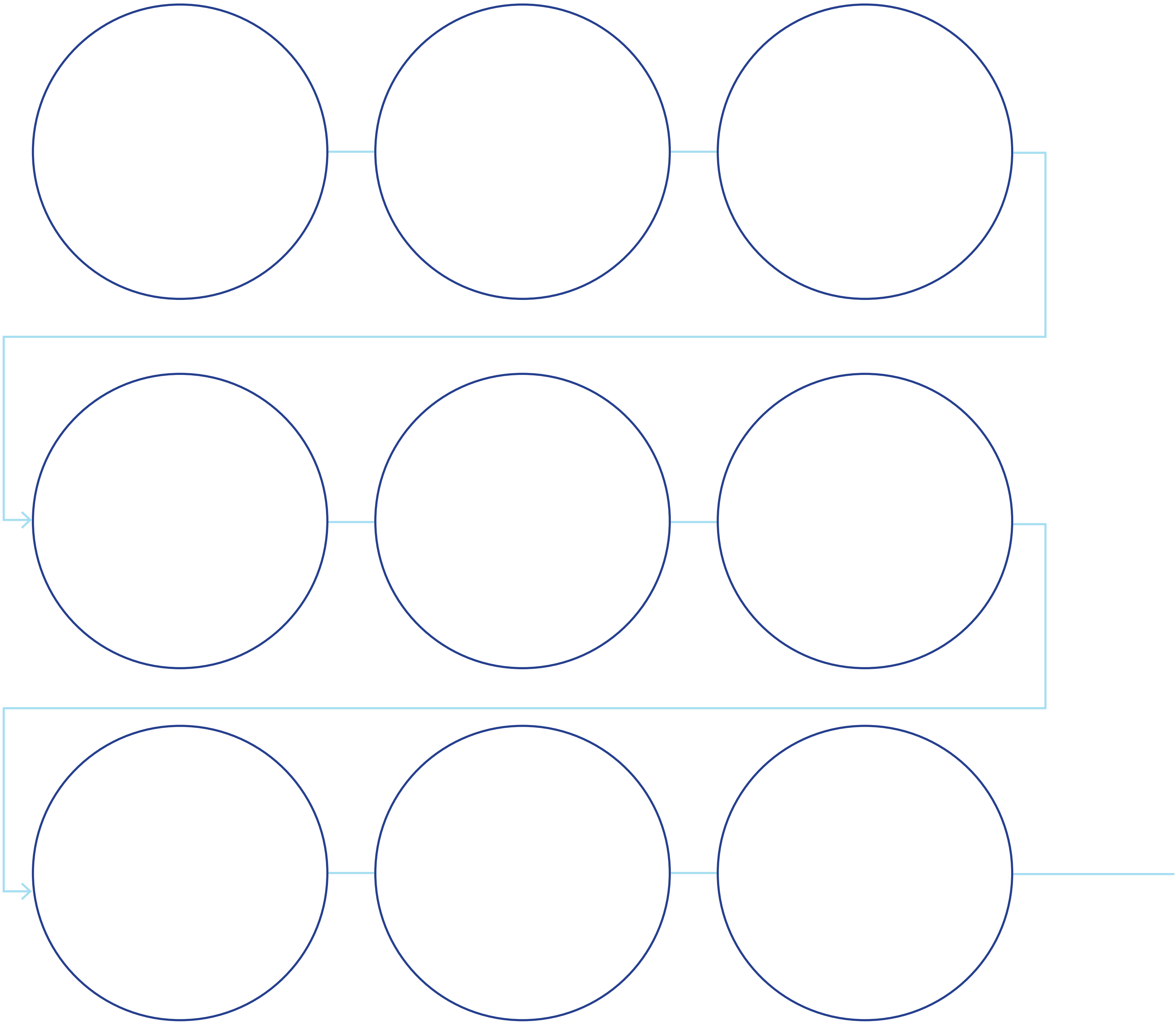

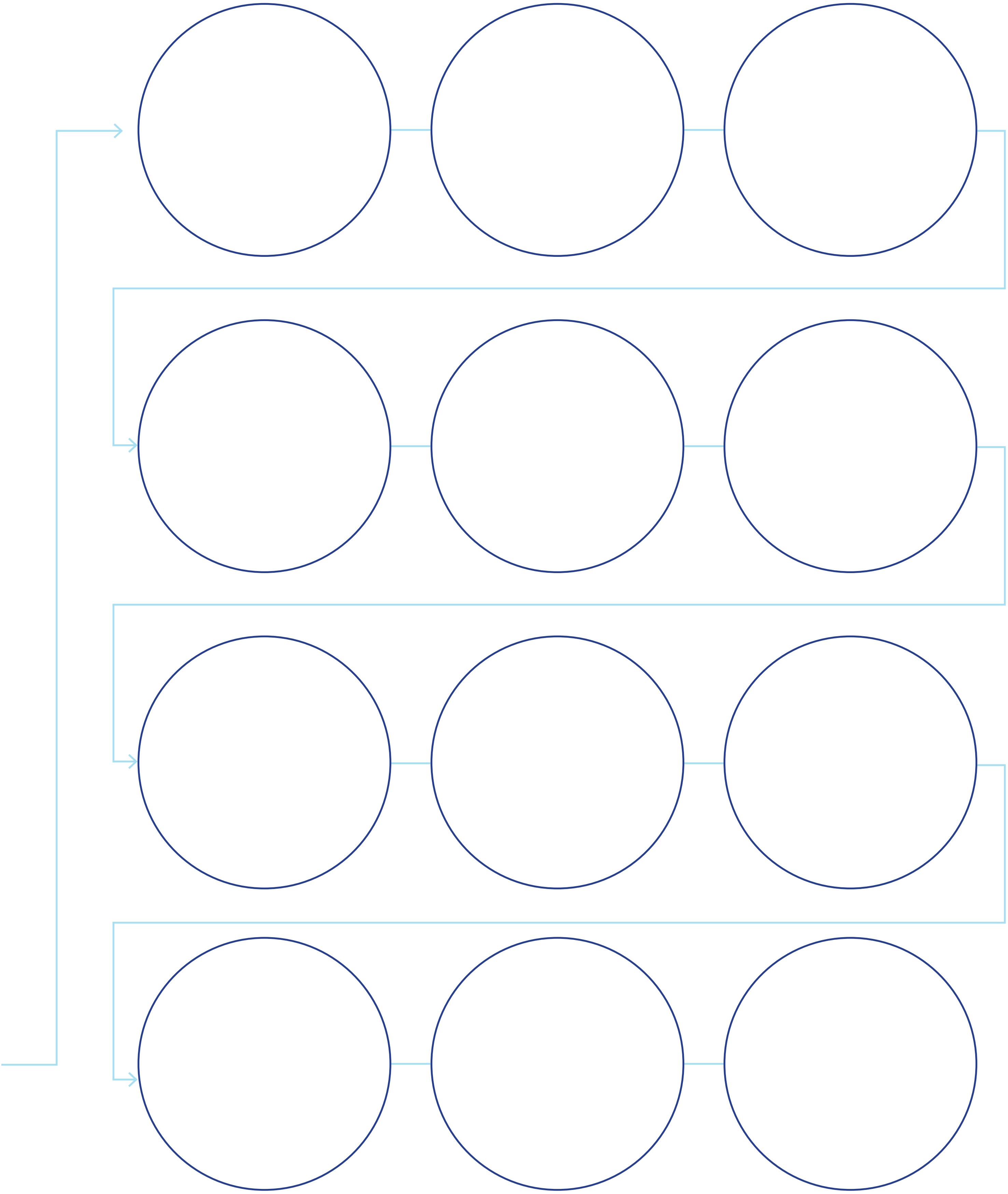

Timeline template

We know that all investigations are unique, so you might like to draw up your own timeline here instead of using the timeline included on the previous page.

Notes

Key words and phrases

Case note review: Use of case or medical notes to determine whether there were any problems with the care provided to a patient, identify the prevalence of issues, or when families/carers or staff raise concerns about care.

Clinical audit: Measurement of the effectiveness of specific elements of healthcare against agreed and proven standards for high quality, with the aim of then acting to bring practice into line with these standards to improve the quality of care and health outcomes.

Clinical Commissioning Group (CCG): CCGs are groups of general practices (GPs) who come together in each area to commission the best services for their patients and the local population. This means that they continuously plan, agree on and monitor NHS services in the local area. **https://www.england.nhs.uk/commissioning/**

Commissioner: Commissioners might include people who have been GPs, or other clinicians such as nurses and consultants. Commissioners will sit on governing bodies which might also include patient representatives, general managers and practice managers.

Common themes: Common themes are recurring ideas, subjects or topics, relevant to the incident and the **Terms of Reference**, the **investigator** identifies when they are reading all of the information they have collected about the incident.

Her Majesty's Coroner: A coroner is a government official or member of the judicial system who carries out **inquests**.

Department for Health and Social Care (DHSC): The government group responsible for health and social care across the UK.

Duty of Candour: A professional responsibility for healthcare staff and organisations to be honest with patients and families when things go wrong. The patient and/or family should be told when something has gone wrong, should be offered an apology and appropriate support, and the full effects of what has happened (if any) should be explained.

Family liaison officer (FLO): A member of Trust staff whose primary role is to provide compassionate support and advice to patients and their families during a **patient safety incident investigation**.

Governance team: Governance teams work in NHS organisations and are responsible for monitoring the quality of services and for safeguarding high standards of care.

Healthcare incident: Any unplanned or unintended event or circumstance which could have resulted or did result in harm to a patient.

The Healthcare Safety Investigation Branch (HSIB): HSIB is funded by the **Department for Health and Social Care**, and is responsible for carrying out independent investigations into NHS-funded care across England.

Hot debrief: A post-incident review by the medical team used to collectively discuss and answer a series of questions.

Incident response lead: A member of staff, normally employed by the healthcare provider, who has been trained to conduct **patient safety incident investigations**. For example, this might be the Trust **patient safety specialist**.

Inquest: An inquest is a formal investigation conducted by a **coroner** to determine how someone died.

Learning disabilities mortality review (LeDeR): A specialist review of the care of a person with a learning disability (recommended alongside a **case note review**).

Legal team: Most NHS Trusts have legal teams to manage a wide range of legal matters for the Trust including: claims brought against the Trust; **inquests**; any proceedings involving Trust witnesses; medical treatment applications to the High Court; medical records requests from solicitors.

Mortality review: A review of a series of case records to identify any problems in care and draw learning or conclusions that inform action needed to improve care, within a setting or for a specific patient group, particularly in relation to deceased patients.

Near miss: An event that does not cause harm, but which has the potential to cause injury or ill health if it had not been caught in time.

Never event: Serious, largely preventable patient safety incidents that should not occur if the available preventative measures have been implemented.

Patient safety incident: Any unintended or unexpected incident which could have, or did, lead to harm for one or more patients receiving healthcare.

Patient safety incident investigation: These are investigations conducted to identify how and why certain patient safety incidents happen. They are not inquiries into the cause of death or for apportioning blame. Investigations result in a set of recommendations and an improvement plan that is designed to effectively and sustainably address the underlying factors in the organisation that led to a patient safety incident, to help deliver safer care in the future.

Patient safety specialist: Individuals in healthcare organisations who have been designated to provide senior patient safety leadership.

Patient safety team: Most NHS Trusts will have a patient safety team dedicated to working within the service to minimise the risk and impact of incidents.

Perinatal mortality review: A specialist multidisciplinary audit and review to determine the circumstances and care leading up to and surrounding a stillbirth or neonatal death, and the deaths of babies in the postneonatal period having received neonatal care.

Policy: An official document that includes a set of guidelines to guide decisions and achieve specific outcomes.

Policy makers: In this context, we mean members of staff from the **Patient safety team** at NHS England and Improvement.

Public inquiry: Public inquiries are independent, national level investigations ordered by a government department to deal with matters of public concern.

Terms of Reference: These are guidelines that define the scope and purpose of the investigation.

This material was developed as part of the Patient and Family Involvement in Serious Incident Investigations (PFI SII) research study. The materials were co-designed by the PFI SII research team, Lab4Living and a community of patients, families, healthcare staff and managers.

FUNDED BY

This study is funded by the National Institute for Health Research NIHR (Health Services and Delivery Research; NIHR Reference Number 18/10/02). The views expressed are those of the authors and not necessarily those of the NIHR or the Department of Health and Social Care.
